# 3D CRANI, a novel MR neurography sequence, can reliable visualise the extraforaminal cranial and occipital nerves

**DOI:** 10.1007/s00330-022-09269-2

**Published:** 2022-11-26

**Authors:** Jan Casselman, Fréderic Van der Cruyssen, Frédéric Vanhove, Ronald Peeters, Robert Hermans, Constantinus Politis, Reinhilde Jacobs

**Affiliations:** 1 Department of Radiology, AZ St-Jan Brugge-Oostende, Ruddershove 10, 8000 Bruges, Belgium; 2 Department of Radiology, AZ St-Augustinus, Antwerp, Belgium; 3 University Ghent, Ghent, Belgium; 4 Department of Oral & Maxillofacial Surgery, University Hospitals Leuven, Kapucijnenvoer 33, 3000 Leuven, Belgium; 5 Department of Imaging and Pathology, OMFS-IMPATH Research Group, Faculty of Medicine, University Leuven, Leuven, Belgium; 6 Department of Radiology, University Hospitals Leuven, Leuven, Belgium; 7 Department of Radiology, AZ Groeninge, Kortrijk, Belgium; 8 Department of Oral Health Sciences, KU Leuven and Department of Dentistry, University Hospitals Leuven, Leuven, Belgium; 9 Department of Dental Medicine, Karolinska Institutet, Stockholm, Sweden

**Keywords:** Magnetic resonance imaging, Neuroimaging, Cranial nerves

## Abstract

**Objectives:**

We aim to validate 3D CRANI, a novel high-field STIR TSE, MR neurography sequence in the visualisation of the extraforaminal cranial and occipital nerve branches on a 3-T system. Furthermore, we wish to evaluate the role of gadolinium administration and calculate nerve benchmark values for future reference.

**Methods:**

Eleven consecutive patients underwent MR imaging including the 3D CRANI sequence before and immediately after intravenous gadolinium administration. Two observers rated suppression quality and nerve visualisation using Likert scales before and after contrast administration. Extraforaminal cranial and occipital nerves were assessed. Nerve calibers and signal intensities were measured at predefined anatomical landmarks, and apparent signal intensity ratios were calculated.

**Results:**

The assessed segments of the cranial and occipital nerves could be identified in most cases. The overall intrarater agreement was 79.2% and interrater agreement was 82.7% (intrarater *κ* = .561, *p* < .0001; interrater *κ* = .642, *p* < .0001). After contrast administration, this significantly improved to an intrarater agreement of 92.7% and interrater agreement of 93.6% (intrarater *κ* = .688, *p* < .0001; interrater *κ* = .727, *p* < .0001).

Contrast administration improved suppression quality and significant changes in nerve caliber and signal intensity measurements. Nerve diameter and signal intensity benchmarking values were obtained.

**Conclusion:**

3D CRANI is reliable for the visualization of the extraforaminal cranial and occipital nerves. Intravenous gadolinium significantly improves MR neurography when applying this sequence. Benchmarking data are published to allow future assessment of the 3D CRANI sequence in patients with pathology of the extraforaminal cranial and occipital nerves.

**Key Points:**

*• MR neurography using the 3D CRANI sequence is a reliable method to evaluate the extraforaminal cranial and occipital nerves.*

*• Gadolinium contrast administration significantly improves suppression quality and nerve visualisation.*

*• Benchmarking values including apparent signal intensity ratios and nerve calibers depend on contrast administration and might play an important role in future studies evaluating extraforaminal cranial and occipital neuropathies.*

**Supplementary Information:**

The online version contains supplementary material available at 10.1007/s00330-022-09269-2.

## Introduction

MR neurography (MRN) in the head and neck region is attracting increasing attention in the literature [1]. This novel MRI technique already showed promise to diagnose peripheral and trigeminal neuropathies [2–4]. MRN may localize the neuropathy and even grade the severity of these neuropathies [5]. The obtained information can be useful in diagnosing and treatment planning of patients with neuropathies. Given the recent introduction of MRN in the head and neck area, only a limited number of validation studies are available. The studies by Chabbra and by Burian illustrated the feasibility of MR neurography of the mandibular nerve and its terminal branches [6, 7]. But no studies are available that validate MR neurography for all extraforaminal cranial or occipital nerves. The purpose of this study was to validate the use of the previously published 3D CRANI (CRAnial Nerve Imaging) [8], a novel high-field STIR TSE, sequence in extraforaminal cranial and occipital nerve visualisation on a 3-T system. Secondary aims were to assess the role of gadolinium administration on imaging quality and to obtain benchmarking values of signal intensities, apparent signal-to-noise (aSNR) and apparent nerve-muscle contrast-to-noise ratios (aNMCNR), and nerve diameters for the evaluated nerve branches.

## Materials and methods

### Subjects

This study was conducted according to the Guidelines for Reporting Reliability and Agreement Studies (GRASS) [9], and additionally, we adhered to the STROBE checklist for observational studies [10]. Retrospectively, 3D CRANI sequencing data was retrieved from consecutive patients visiting the radiology department of Bruges, Belgium, and who underwent head and neck MR imaging. Patients were included whenever the senior radiologist (J.C.) could not identify pathology along the extraforaminal cranial and occipital nerve branches and when a 3D CRANI sequence was present before and after gadolinium contrast administration. Thus, no pathology was present along the course of the observed nerve branches on both sides. Moreover, none of the patients received radiotherapy in the head and neck area nor did they receive chemotherapy. The reason for MRI referral is addressed in supplemental Table 1. Ethical committee approval was waived due to the retrospective nature of this study.

### MRI Imaging procedure

Imaging was performed on a 3.0-Tesla (T) MRI system (Ingenia; Philips) equipped with 32 channel head coil (INVIVO). A previously published MR neurography sequence, 3D CRANI, was performed [1, 8]. 3D CRANI is a 3D TSE STIR sequence that uses a PSS (pseudo-steady state) sweep in combination with MSDE (Motion Sensitized Driven Equilibrium) Pulse. We used STIR in combination with MSDE to ensure the signal from fat, muscle, and blood is suppressed uniformly across the field of view.

The following parameters were applied: TR = 2300 ms, TE = 188 ms, FOV = 200 × 200 × 90 mm, slice thickness = 0.9 mm, act slice gap = −0.45 mm, matrix = 224 × 222 mm, acquired voxel size = 0.9 × 0.9 × 0.9 mm, reconstructed voxel Size = 0.6 × 0.6 × 0.45 mm, slice oversampling = 1.5, compressed sense, (reduction 2), number of slices = 200. TSE Nerve STIR, TSE factor = 43 (startup echoes 2), number of acquisitions = 1, scanning time 8:08 min, BB pulse = MSDE (flow ghost suppression). The 3D CRANI sequence was repeated immediately after the administration of gadolinium.

### Imaging analysis

**Fig. 1:**
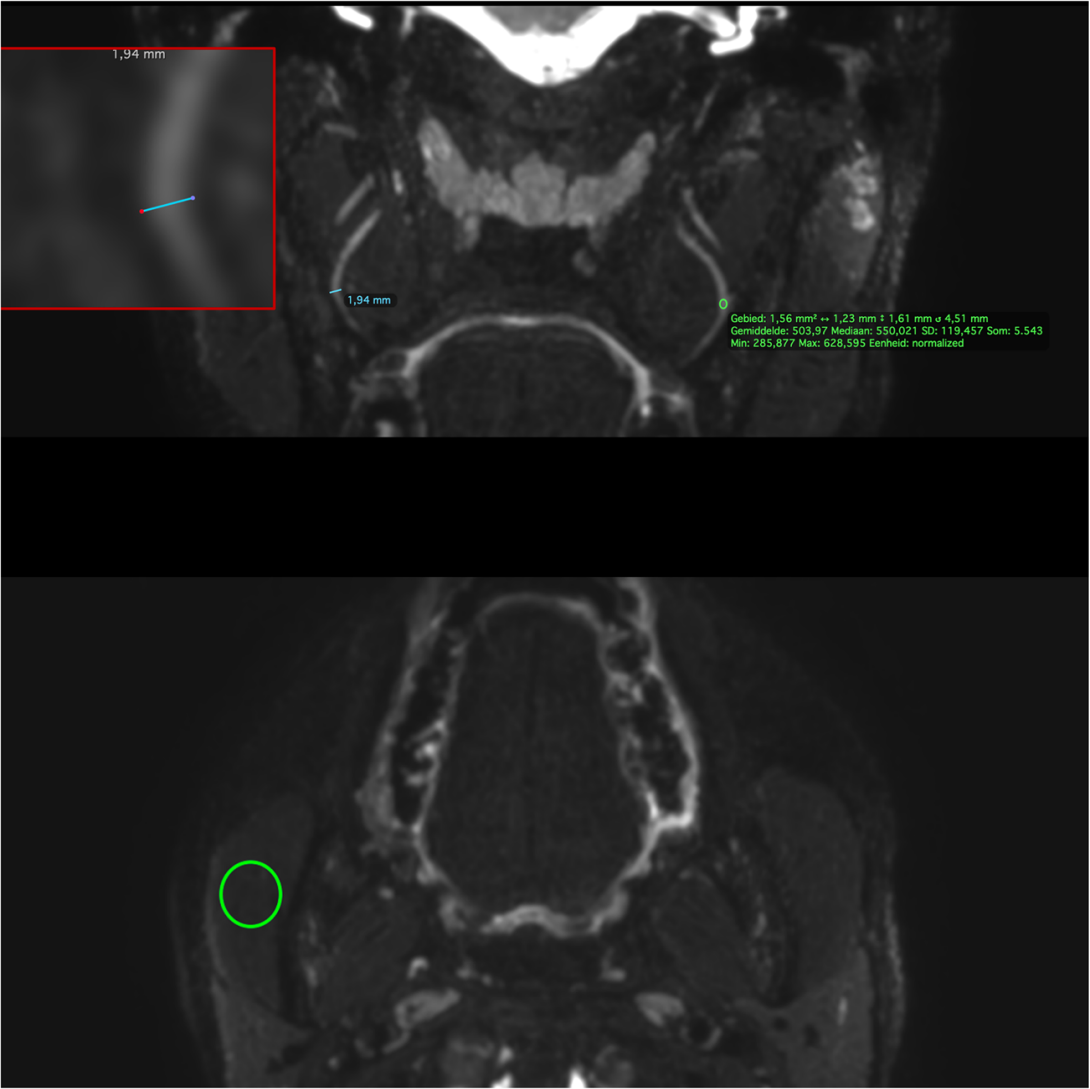
ROI measurements on the 3D CRANI sequence of the midpoint of the lingual nerve. Using the magnifying tool (red box at top inset) the nerve diameter (blue ROI line) can be accurately measured in a coronal view. To measure signal intensity, a ROI is placed at predefined landmarks within the nerve contour (upper green ROI circle). A 1 cm^2^ ROI circle is used to measure muscle signal intensity in an axial view (right masseter muscle: lower green ROI circle) and air signal intensity within the right maxillary sinus (not illustrated here)

Three orthogonal planes, as well as a plane following the course of the mandibular nerve using multiplanar reformation (MPR) and maximum intensity projection (MIP), were reconstructed using the Philips Volume post-processing package. A reformatted slab thickness of 5 mm and gap of −0.5 mm allowed for the best demonstration of the nerve trajectory. The images were analyzed by two trained observers (F.V.D.C. with 5 years of experience in head and neck imaging, F.V. with 5 years of radiology experience, and 2 years in head and neck imaging). After a calibration session, initial evaluations were made independently and blinded from each other using a scoring form (Table 1). The observers first scored the suppression quality for arteries, veins, fat, and lymph nodes before and after contrast administration on the 3D CRANI sequence. Next, all cranial nerves were assessed and scored for visualization before and after contrast administration. The following nerves were evaluated on both sides: trigeminal nerve branches, facial nerve, glossopharyngeal nerve, vagus and accessory nerve, hypoglossal nerve, and the greater and lesser occipital nerves. We defined a midpoint for each cranial nerve resulting in a proximal and distal segment (Table 1). Both observers were asked if they could identify each nerve before and after contrast administration. Next, a nerve visualisation score was adopted using a 5-point scale (4, excellent: both proximal and distal portion identified; 3, good: both portions identified but not continuous; 2, fair: only proximal portion identified; 1, poor: only proximal portion identified but not continuous; 0, nerve could not be identified) [11]. If the nerve was not located in the field of view, this could also be indicated. The observers were allowed to consider the proximal portion of cranial nerves IX-X-XI as one and the same given their close anatomical location and in accordance with a previously published study [11]. The measurements were repeated after one month by both observers and after randomizing all cases. After this qualitative analysis, each nerve was analyzed quantitatively to obtain benchmark values before and after contrast administration during the first observation session. Both observers measured signal intensities of the cranial nerves by placing a circular region of interests (ROI) within the identified cranial nerves (iROI) at the predefined landmarks. Similarly, a 1 cm^2^ ROI was drawn within the masseter muscle (mROI) and in air (aROI) (Fig. 1). The apparent signal-to-noise ratio (aSNR), the apparent nerve-muscle contrast-to-noise ratio (aNMCNR) and nerve diameter were measured for each cranial nerve. aSNR and aNMCNR were calculated by normalising with the standard deviation of air (SD_air_) [4]. Equations used to calculate aSNR and aNMCNR:

$$ aSNR=\frac{iROI}{SD_{air}} $$

$$ aNMCNR=\frac{iROI- mROI}{SD_{air}} $$

**Table 1:** Assessment form illustrating qualitative Likert-scales to rate suppression quality and nerve visualization. The landmarks used for the evaluation of suppression quality and calculation of nerve dimensions and signal intensity are also listed

Suppression quality score				
1	Not suppressed, not diagnostically usable			
2	Not suppressed, but diagnostically usable			
3	Moderately suppressed, diagnostically usable			
4	Excellent suppression, diagnostically usable			
Suppression quality landmarks				
Arterial	Internal carotid artery			
Venous	Pterygoid plexus			
Fat	Subcutaneous fat plane			
Lymph nodes	Lymph nodes in neck level II/III			
Nerve identification				
0	Not identified			
1	Identified			
Nerve visualisation score				
0	Nerve not identified			
1	Poor—only proximal portion identified but not continuous			
2	Fair—only proximal portion identified			
3	Good fair—both portions identified but not continuous			
4	Excellent—both proximal and distal portion identified			
99	Nerve not within field of view			
Nerve landmarks				
	Proximal	Midpoint	Distal	Viewing plane for evaluation
V1 Opthalmic nerve	Meckel’s cave	Entry of orbit	Supraorbital rim	Axial
V2 Infraorbital nerve	Meckel’s cave	Posterior wall of maxillary sinus	Infraorbital foramen	Axial
V3 Inferior alveolar nerve	Skullbase	Mandibular foramen	Mental foramen	Coronal oblique
V3 Lingual nerve	Skullbase	Maximum convex point	Entry of base of tongue	Coronal oblique
V3 Buccal nerve	Skull base	Maximum convex point	Entry of buccinator muscle	Axial
V3 Masseteric nerve	Skull base	Medial border of lateral pterygoid muscle	Entry of masseter muscle	Axial
V3 Deep temporal nerve	Skull base	Medial border of lateral pterygoid muscle	Entry of temporal muscle	Axial
V3 Auriculotemporal nerve	Skull base	Midway between skull base and TMJ	Medial condylar surface	Axial
VII Facial nerve	Stylomastoid foramen	Entry of parotid gland	Exit of parotid gland	Coronal
IX Glossopharyngeal nerve	Skull base	Posterior wall of carotid	Pharyngeal wall	Coronal
X Vagus nerve	Skull base	Posterior wall of carotid	Exit of field-of-view	Coronal
XI Accessory nerve	Skull base	Posterior wall of carotid	Trapezius muscle	Coronal
XII hypoglosal nerve	Skull base	Posterior wall of carotid	Anterior border of submandibular gland	Coronal/Axial
Greater occipital nerve	Cervical vertebrae	Semispinal muscle	Trapezius muscle	Axial
Lesser occipital nerve	Cervical vertebrae	Obliquus capitis inferior muscle	Skin	Axial

### Statistical analysis

All statistical analyses were done by a certified statistician (FVDC) with RStudio Team (2020) (RStudio: Integrated Development for R. RStudio, PBC). Descriptive statistics were carried out after pooling of left and right sides as scored by the observers. Confidence intervals of 95% were calculated where suited. A Pearson chi-squared test was used to assess the independence of nerve identification and suppression quality scores and Fleiss’ kappa statistics to assess inter- and intrarater agreement on the ordinal outcome measures (nerve identification and suppression quality). Group differences between continuous measurements were compared using a Student’s T-test or ANOVA test in the case of multiple groups. Intraclass correlation coefficients were calculated to determine agreement on the quantitative continuous measurements. A *p* value of less than 0.05 was considered significant. There was no missing data in the final dataset.

## Results

### Nerve identification and visualisation score

**Fig. 2:**
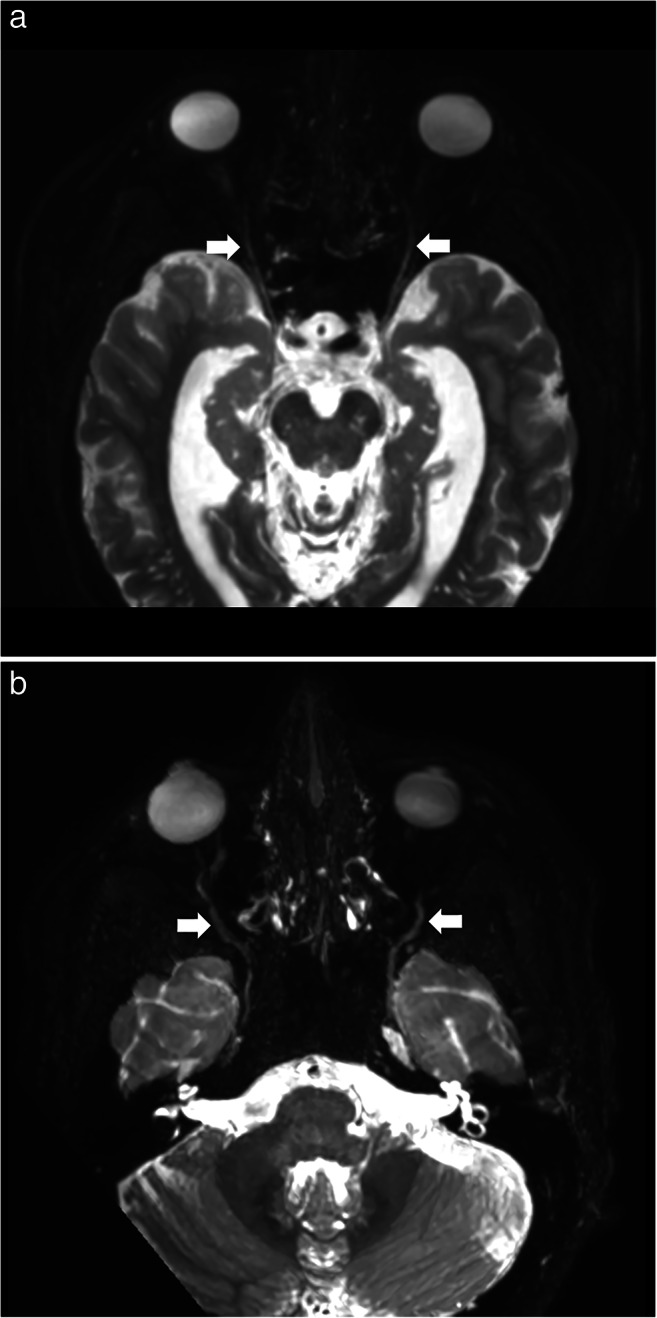
**a** Axial view of the 3D CRANI sequence immediately after contrast administration illustrating the ophthalmic division of the trigeminal nerve (white arrows) entering the orbit. **b** Axial view of the 3D CRANI sequence immediately after contrast administration illustrating the maxillary nerve (second division of the trigeminal nerve) starting at Meckel’s cave and its infraorbital branch coursing inferior to the optic nerve towards the infraorbital foramen

**Fig. 3:**
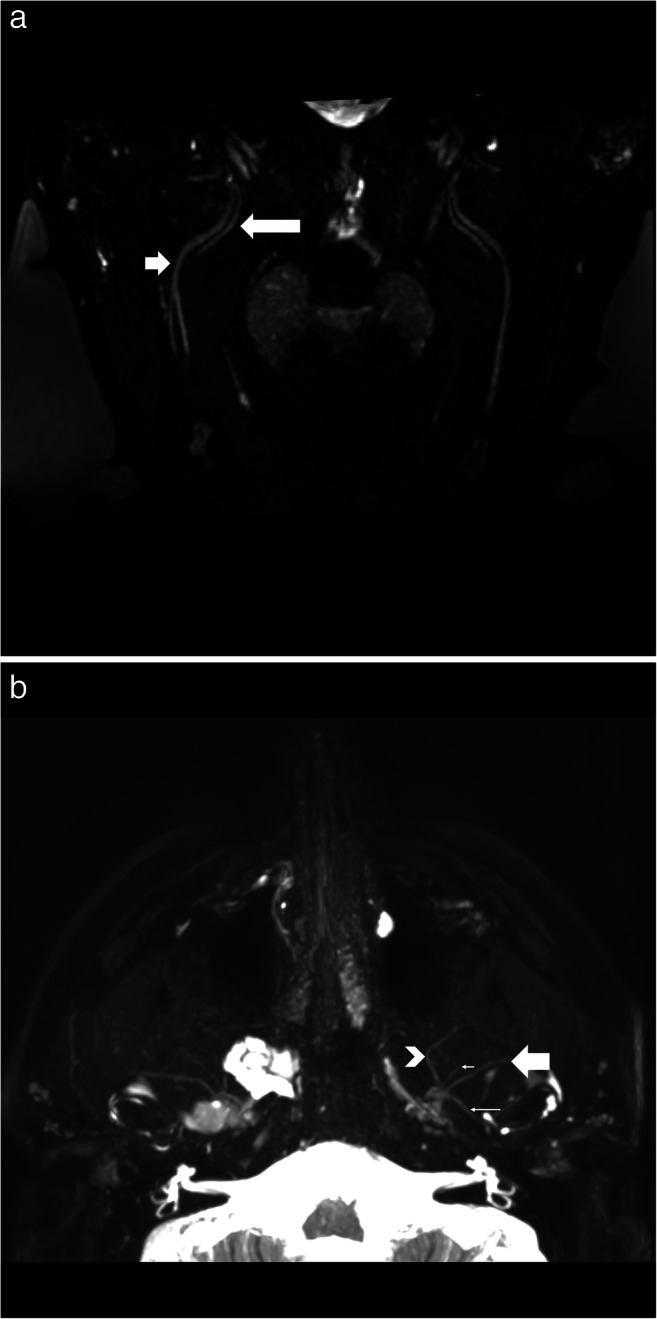
**a** Oblique coronal view of the 3D CRANI sequence immediately after contrast administration illustrating the lingual nerve (long arrow) and inferior alveolar nerve (short arrow) running lateral to the pterygoid muscles on an oblique coronal viewing plane. Barium filled bags were used to fixate the patient’s head and further improve the suppression quality of surrounding tissues. **b** Third division of the trigeminal nerve in an axial view. This illustrates the ability of the 3D CRANI sequence to visualise the buccal (arrowhead), deep temporal (small short arrow), auriculotemporal (small long arrow), and masseteric (large arrow) nerves

**Fig. 4:**
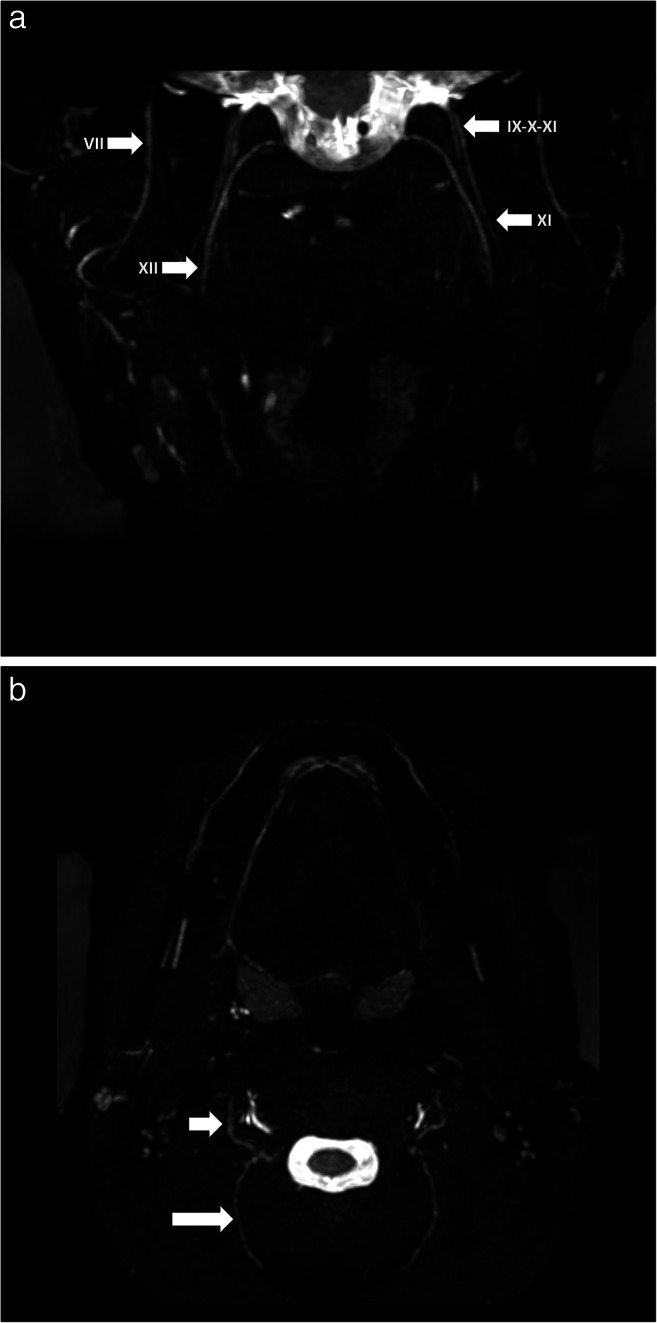
**a** Visualization of facial (VII), hypoglossal (XII), accessory (XI) and glossopharyngeal-vagus (IX-X) nerves on a coronal 3D CRANI sequence immediately after contrast administration. **b** Greater occipital (long arrow) and lesser occipital nerves on an axial 3D CRANI viewing plane

**Fig. 5:**
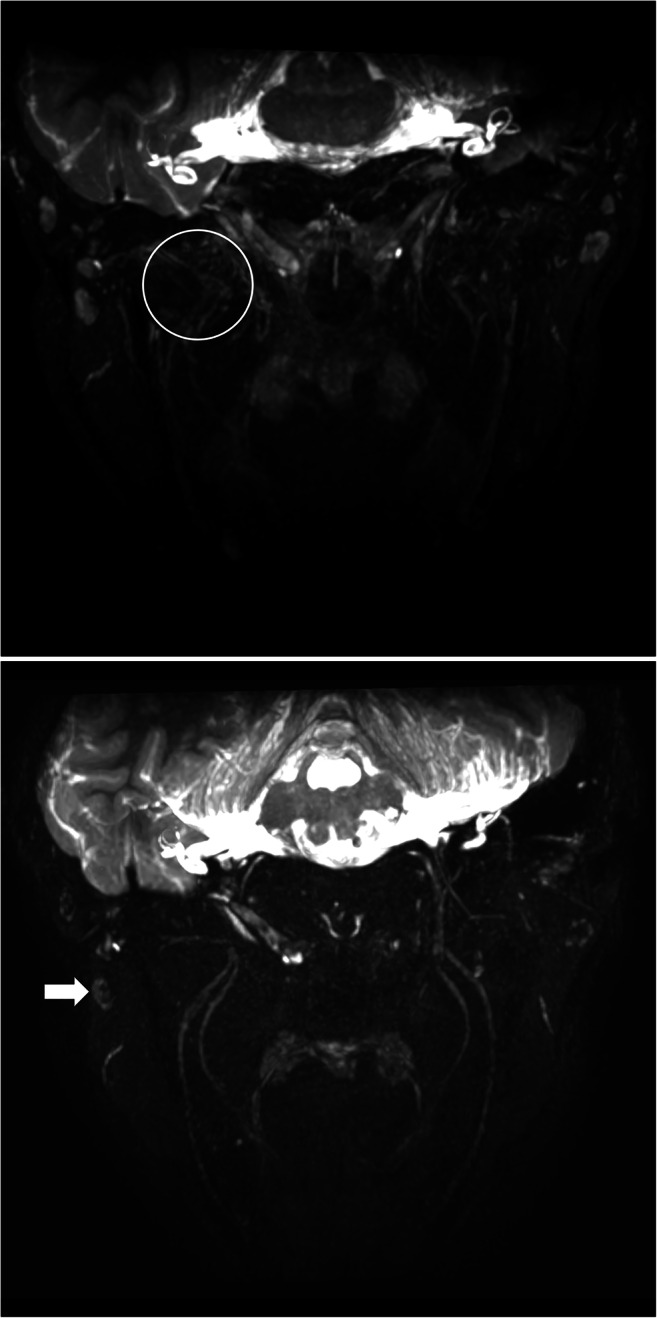
**a** Venous plexus artefacts before contrast administration limiting the visualization of the third division of the trigeminal nerve in the area of the pterygoid muscles and plexus. **b** Same patient as in Figs. 1, 2, 3, 4 and 5 after gadolinium contrast administration. Remarkable improvement in suppression quality and nerve visualisation. Some lymph nodes remain poorly suppressed (white arrow)

**Fig. 6:**
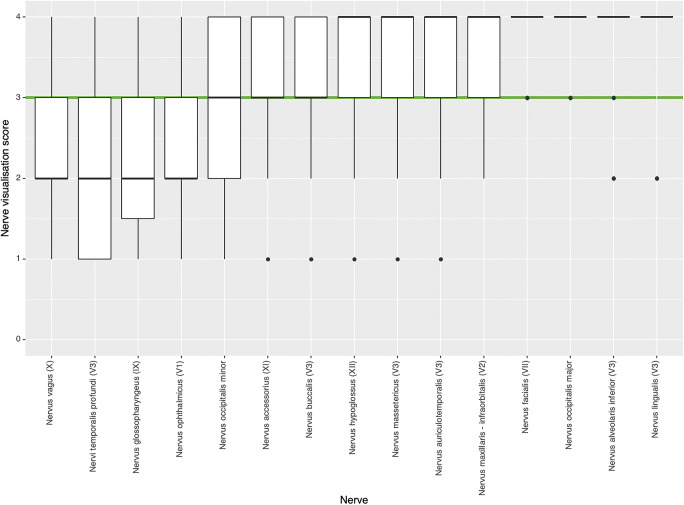
Qualitative nerve visualisation scores as assessed by both observers using a 5-point scale (4, excellent: both proximal and distal portion identified; 3, good: both portions identified but not continuous; 2, fair: only proximal portion identified; 1, poor: only proximal portion identified but not continuous; 0, nerve could not be identified). Most nerves were rated as good to excellent visualization (green cut-off line)

Data from eleven patients were included in this study between January and September 2020 (Supplemental table 1): six males and five females with an average age of 47 (range: 14–83). Most extraforaminal cranial nerve branches could be identified in all subjects by both observers after administration of gadolinium contrast agent, except for the lesser occipital and ophthalmic division of the trigeminal nerve where detection rates were considerably lower (Table 2). The use of gadolinium contrast significantly improved nerve detection rates on the 3D CRANI sequence when comparing combined detection rates before and after contrast administration (*p* < 0.001). 3D CRANI allowed us to obtain high spatial resolution (Figs. 2, 3, 4, and 5). The ophthalmic trigeminal branch and the occipital nerve branches were the most difficult to distinguish as illustrated by lower identification scores. A similar pattern was seen when nerve visualisation scores were evaluated (Fig. 6). On average, the visualisation of most cranial nerve branches was scored as good to excellent, except for the glossopharyngeal and vagus nerves and the smaller nerve branches such as the deep temporal and ophthalmic nerves which still received a fair score meaning the proximal portion of these branches could be identified. Nerve identification before contrast administration showed an overall intrarater agreement of 79.2% and interrater agreement of 82.7% (intrarater *κ* = .561, *p* < .0001; interrater *κ* = .642, *p* < .0001). After contrast administration, this improved to an overall intrarater agreement of 92.7% and interrater agreement of 93.6% (intrarater *κ* = .688, *p* < .0001; interrater *κ* = .727, *p* < .0001).

**Table 2:** Nerve identification scores (nerve identified: yes or no) as assessed by both observers before and after contrast administration. This is expressed as a percentage where one hundred percent means that the nerve could be detected in all cases. A significant improvement in detection rates is established after contrast administration

Percentage detected (%)	Without Gd contrast	With Gd contrast
Nervus ophthalmicus (V1)	29.5	36
Nervus maxillaris - infraorbitalis (V2)	98.9	100
Nervus alveolaris inferior (V3)	100	100
Nervus lingualis (V3)	100	100
Nervus buccalis (V3)	38.6	100
Nervus auriculotemporalis (V3)	28.4	97.7
Nervus massetericus (V3)	37.5	96.6
Nervi temporalis profundi (V3)	8	72.7
Nervus facialis (VII)	100	100
Nervus glossopharyngeus (IX)	43.2	89.8
Nervus vagus (X)	51.1	85.2
Nervus accessorius (XI)	75.9	94.3
Nervus hypoglossus (XII)	88.5	95.5
Nervus occipitalis major	70.8	72.7
Nervus occipitalis minor	54.5	56.8

Pearson’s chi-squared test, *p* < 0.001; *Gd*, gadolinium

### Suppression quality of surrounding structures

The arterial and fat suppression quality was moderate to excellent both before and after contrast administration. Venous and lymph node suppression quality was scored non-suppressed to excellently suppressed, with an improvement in suppression quality after contrast administration (Table 3, Fig. 5). Excellent agreement was seen for arterial and fat suppression. Venous and lymph node suppression quality scores showed varying agreement between and within observers. Kappa statistics varied from poor to moderate (Supplemental table 2).

**Table 3:** Suppression quality scores before and after contrast administration. A significant improvement in suppression quality is seen after contrast administration. Lymph nodes remain not too moderately suppressed immediately after contrast administration

Suppression quality score	Without Gd contrast	With Gd contrast
Arterial		
1: Not suppressed, not diagnostically usable	0 (0%)	0 (0%)
2: Not suppressed, but diagnostically usable	0 (0%)	0 (0%)
3: Moderately suppressed, diagnostically usable	0 (0%)	0 (0%)
4: Excellent suppression, diagnostically usable	44 (100%)	44 (100%)
Venous		
1: Not suppressed, not diagnostically usable	1 (2%)	0 (0%)
2: Not suppressed, but diagnostically usable	20 (46%)	0 (0%)
3: Moderately suppressed, diagnostically usable	22 (50%)	14 (32%)
4: Excellent suppression, diagnostically usable	1 (2%)	30 (68%)
Fat tissue		
1: Not suppressed, not diagnostically usable	0 (0%)	0 (0%)
2: Not suppressed, but diagnostically usable	0 (0%)	0 (0%)
3: Moderately suppressed, diagnostically usable	18 (41%)	3 (7%)
4: Excellent suppression, diagnostically usable	26 (59%)	41 (93%)
Lymphatic tissue		
1: Not suppressed, not diagnostically usable	0 (0%)	0 (0%)
2: Not suppressed, but diagnostically usable	38 (86%)	15 (34%)
3: Moderately suppressed, diagnostically usable	4 (9%)	26 (59%)
4: Excellent suppression, diagnostically usable	2 (5%)	3 (7%)

Pearson’s chi-squared test, *p* < 0.001; *Gd*, gadolinium

### Quantitative analysis: benchmarking values and reliability

Nerve benchmarking values were calculated before and after contrast administration (Supplemental table 3). Excellent aSNR (M = 36.2, SD = 14.5) and aNMCNR (M = 24.1, SD = 14.7) were seen along nerve trajectories post contrast administration, with a decrease in aSNR, aNMCR, and diameter from proximal to distal for all nerve branches (Supplemental figures). Nerve branches as small as 0.5 millimeters could be identified. A significant decrease in nerve diameter measurements and aSNR was observed after contrast administration (*p* < .05). aNMCNR did not significantly differ before and after contrast administration. The intraclass correlation coefficients (ICC) showed high concordance for all measurements with decreasing ICC values from proximal to distal (Table 4).

**Table 4.** Intraclass correlation coefficients (ICC) and confidence intervals for quantitative apparent signal-to-noise ratios (aSNR) and nerve-muscle contrast-to-noise-ratios (aNMCNR) before and immediately after contrast administration measured by both observers during the first session.

	Without Gd contrast			With Gd contrast		
	ICC	ICC, lower limit	ICC, upper limit	ICC	ICC, lower limit	ICC, upper limit
aSNR, proximal	0.7346	0.6805	0.7807	0.7316	0.6771	0.7781
aSNR, mid	0.689	0.6277	0.7418	0.6265	0.556	0.688
aSNR, distal	0.6725	0.6086	0.7277	0.5922	0.5173	0.6581
Diameter, proximal	0.773	0.7255	0.8132	0.7144	0.6572	0.7635
Diameter, mid	0.7461	0.6941	0.7904	0.7274	0.6721	0.7746
Diameter, distal	0.71	0.6519	0.7598	0.6503	0.5832	0.7085
aNMCNR, proximal	0.7317	0.6772	0.7783	0.6157	0.5439	0.6786
aNMCNR, mid	0.6165	0.5447	0.6794	0.5734	0.4961	0.6417
aNMCNR, distal	0.6608	0.5952	0.7177	0.4679	0.3791	0.5482

*Gd*, gadolinium; *ICC*, intraclass correlation coefficient; *aSNR*, apparent signal-to-noise ratio*; aNMCNR*, apparent nerve-muscle contrast-to-noise ratio

## Discussion

This study confirms that the novel MR neurography sequence, also denoted as 3D CRANI [8], is a reliable and reproducible MR neurography technique for the visualisation of the extraforaminal cranial and occipital nerves. Previous studies already evaluated the feasibility of heavily T2-weighted MR imaging for nerve-specific visualization of the mandibular nerve [2, 12] but this is the first study to expand on this topic and evaluate the reliability of MRN in cranial and occipital nerve evaluation. Reliable imaging techniques are necessary when dealing with cranial nerve disorders, as electrophysiological and sensory examinations in the head and neck area have their own limitations [13]. Some already described the advantageous role of MRN in diagnosing trigeminal nerve injuries and impact on clinical management [2, 5]. Within other domains such as brachial plexus imaging, MRN established its role and showed substantial therapeutic impact in over one third of patients [14].

This is the first study to assess the role of contrast administration in MR neurography. We illustrated improved suppression quality of surrounding structures as well as improved nerve visualisation after gadolinium administration. This probably results from a short-lasting change in susceptibility of the contrast-filled vessels resulting in faster blood dephasing and thus a better suppression quality.

A significant decrease in signal intensities and nerve diameters immediately after contrast administration was noticed. A possible explanation could be the improved suppression of the surrounding tissues and vasa nervorum. As a result, true MR neurography is achieved. This further implies that benchmarking of signal intensity, but also spatial dimensions, depends on contrast administration. Current literature does not allow unequivocal comparison of benchmarking values as each study applies its own MR sequences, and relative signal calculations, with or without contrast administration [4–6]. One study by Burian et al evaluating the lingual and inferior alveolar nerves did produce similar nerve diameters [6]. However, aSNR and aMNCNR do not seem to correspond. Perhaps because different formulas for signal calculation were applied. Publishing all relevant data may overcome this hurdle for future comparison. Furthermore, future studies could compare pathological nerve thickening found on MRN with surgical findings, as exemplified by the work of Zuniga et al [3].

A signal intensity drop moving from proximal to distal along the nerve trajectory was seen. And, as one would expect, the nerve diameter also decreased in the distal direction. This is an important fact if we want to be able to make statements about pathological abnormalities in cranial and occipital neuropathy in the future. Others found similar signal changes in both healthy volunteers and neuropathy cases [4]. In the case of traumatic neuropathies, an increase in focal signal intensity and caliber correlates with histological changes such as endoneural edema, vascular congestion, onset of endoneural fibrosis, and demyelination [15]. Bendszus and colleagues further identified temporal MR changes in the weeks following sciatic nerve lesions in a rat model that correlated with electrophysiological findings [15].

This study had some limitations including its retrospective nature, a small sample size, and limited number of observers. However, a wide age distribution and near-equal female-male ratio was achieved. Both observers anticipated a calibration session to limit method bias. Future studies should confirm these findings on a larger cohort. A large number of measurements could have resulted in measurement errors. Automatic segmentation and signal intensity calculation would be a next step forward in determining benchmarking values for any anatomical location, limiting this bias. The occipital nerves showed a surprisingly low overall visualisation score, probably this was related due to patient positioning resulting in suboptimal suppression quality in the occipital area and not due to inherent flaws in the MRN technique; however, this must be verified in a future study. Suppression quality scores showed varying results both between and within observers. This could be due to several factors such as the use of a limited 4-point Likert scale to score suppression quality and small sample size. Finally, a case-control study will be needed to address the reliability of 3D CRANI in patients with cranial or occipital nerve disorders.

## Conclusion

This study confirms the reliability of the novel 3D CRANI sequence for MR neurography of the extraforaminal cranial and occipital nerves in healthy subjects. Intravenous gadolinium administration improves suppression quality and nerve visualisation but alters signal intensities and nerve calibers. Quantitative measurements are reproducible and may serve as benchmarking for future case-control studies on cranial nerve disorders.

## Supplementary information

ESM 1 (DOCX 1963 kb)

## Abbreviations

3D: Three dimensional
aNMCNR: Apparent nerve-muscle contrast-to-noise ratio
ANOVA: Analysis of variance
aSNR: Apparent signal-to-noise ratio
CRANI: CRAnial Nerve Imaging
FOV: Field of view
GRASS: Guidelines for Reporting Reliability and Agreement Studies
iROI: Region of interest measured within observed nerve
MIP: Maximum intensity projection
MPR: Multiplanar reformation
MRN: Magnetic resonance neurography
mROI: Region of interest measured at the masseter muscle
MSDE: Motion Sensitized Driven Equilibrium
PSS: Pseudo-steady state
SDair: Standard deviation of signal intensity measured within air
STIR TSE: Short TI inversion recovery turbo spin echo
STROBE: STrengthening the Reporting of OBservational studies in Epidemiology.
T: Tesla
TE: Echo time
TR: Repetition time

## Declarations

### Guarantor

The scientific guarantor of this publication is Fréderic Van der Cruyssen.

### Statistics and biometry

One of the authors has significant statistical expertise.

### Methodology

• retrospective

• observational

• performed at one institution
