# Supplementary material for: 3D CRANI, a novel MR neurography sequence, can reliable visualise the extraforaminal cranial and occipital nerves
Source: Eur Radiol. 2022 Nov 26;33(4):2861–70. doi: 10.1007/s00330-022-09269-2 (PMC10017653; doi:10.1007/s00330-022-09269-2)

| **ID** | **Gender** | **Age** | **Reason for MRI referral** |
| --- | --- | --- | --- |
| 1 | M | 65 | Suspected trigeminal neuralgia |
| 2 | F | 57 | Suspected trigeminal neuralgia |
| 3 | M | 53 | Persistent tension headache |
| 4 | M | 49 | Suspected trigeminal neuralgia |
| 5 | M | 24 | Suspected occipital neuralgia |
| 6 | F | 55 | Infraorbital weakness after facelift |
| 7 | M | 66 | Suspected parotid tumor |
| 8 | F | 35 | Pineal cyst |
| 9 | F | 14 | Inexplainable sudden drop attack |
| 10 | M | 83 | Hearing loss |
| 11 | F | 16 | Suspected lingual nerve deficit |

Supplemental table 1. Patient characteristics and reason for MRI referral.

|  | **Contrast administration** | **% agreement, inter-rater** | **Inter-rater kappa** | **Inter-rater kappa, CI** | **% agreement, rater 1** | **Intra-rater kappa rater 1** | **Intra-rater rater1 kappa, CI** | **% agreement, rater 2** | **Intra-rater kappa rater 2** | **Intra-rater rater2 kappa, CI** | **% agreement, rater 1 and 2** | **Intra-rater kappa, rater 1 and 2** | **Intra-rater kappa, rater 1 and 2, CI** |
| --- | --- | --- | --- | --- | --- | --- | --- | --- | --- | --- | --- | --- | --- |
| **Arterial** | No | 1 | 1 |  | 1 | 1 |  | 1 | 1 |  | 1 | 1 | 1 |
| **Arterial** | Yes | 1 | 1 |  | 1 | 1 |  | 1 | 1 |  | 1 | 1 | 1 |
| **Venous** | No | 0.5 | 0.1619 | [-0.207;0.5308] | 0.7 | 0.4054 | [-0.1094;0.9202] | 0.4 | -0.2941 | [-0.8132;0.225] | 0.5 | 0.1619 | [-0.207;0.5308] |
| **Venous** | Yes | 0.8 | 0.581 | [0.1631;0.9989] | 0.5 | -0.0476 | [-0.6386;0.5434] | 0.7 | 0.3714 | [-0.2196;0.9624] | 0.6 | 0.1619 | [-0.256;0.5798] |
| **Fat tissue** | No | 0.8 | 0.6239 | [0.206;1.0418] | 0.3 | -0.5714 | [-1.1624;0.0196] | 0.5 | -0.1 | [-0.691;0.491] | 0.4 | -0.3162 | [-0.7341;0.1017] |
| **Fat tissue** | Yes | 0.9 | -0.05 | [-0.4777;0.3777] | 0.9 | -0.0526 | [-0.6724;0.5672] | 0.9 | -0.0476 | [-0.6386;0.5434] | 0.9 | -0.05 | [-0.4777;0.3777] |
| **Lymphatic tissue** | No | 0.7 | -0.1186 | [-0.4528;0.2156] | 0.7 | 0.12 | [-0.3685;0.6085] | 0.8 | -0.0732 | [-0.5338;0.3874] | 0.8 | 0.0678 | [-0.2664;0.402] |
| **Lymphatic tissue** | Yes | 0.8 | 0.5712 | [0.2199;0.9225] | 0.5 | 0.12 | [-0.402;0.642] | 0.5 | 0.1603 | [-0.3162;0.6368] | 0.5 | 0.1423 | [-0.209;0.4936] |
| CI: confidence interval | | | | | | | | | | | | | |

Supplemental table 2. Suppression quality inter- and intrarater agreement and kappa statistics before and immediately after contrast administration.

|  | **Nervi temporalis profundi (V3)**, N = 88 | **Nervus accessorius (XI)**, N = 88 | **Nervus alveolaris inferior (V3)**, N = 88 | **Nervus auriculotemporalis (V3)**, N = 88 | **Nervus buccalis (V3)**, N = 88 | **Nervus facialis (VII)**, N = 88 | **Nervus glossopharyngeus (IX)**, N = 88 | **Nervus hypoglossus (XII)**, N = 88 | **Nervus lingualis (V3)**, N = 87 | **Nervus massetericus (V3)**, N = 88 | **Nervus maxillaris - infraorbitalis (V2)**, N = 88 | **Nervus occipitalis major**, N = 88 | **Nervus occipitalis minor**, N = 88 | **Nervus ophthalmicus (V1)**, N = 89 | **Nervus vagus (X)**, N = 88 |
| --- | --- | --- | --- | --- | --- | --- | --- | --- | --- | --- | --- | --- | --- | --- | --- |
| **Before gadolinium contrast administration** | | | | | | | | | | | | | | | |
| **Nerve visualisation score** |  |  |  |  |  |  |  |  |  |  |  |  |  |  |  |
| 0 Nerve not identified | 81 (92%) | 14 (16%) | 0 (0%) | 63 (72%) | 54 (61%) | 0 (0%) | 44 (50%) | 3 (3.4%) | 0 (0%) | 55 (62%) | 0 (0%) | 1 (1.1%) | 15 (17%) | 1 (1.1%) | 37 (42%) |
| 1 Poor - Only proximal portion identified but not continuous | 5 (5.7%) | 31 (36%) | 0 (0%) | 12 (14%) | 21 (24%) | 9 (10%) | 21 (24%) | 27 (31%) | 2 (2.3%) | 11 (12%) | 2 (2.3%) | 2 (2.2%) | 11 (12%) | 11 (12%) | 21 (24%) |
| 2 Fair - Only proximal portion identified | 0 (0%) | 8 (9.2%) | 6 (6.9%) | 7 (8.0%) | 9 (10%) | 21 (24%) | 14 (16%) | 29 (33%) | 18 (21%) | 8 (9.1%) | 20 (23%) | 7 (7.9%) | 17 (19%) | 13 (15%) | 18 (20%) |
| 3 Good Fair - Both portions identified but not continuous | 2 (2.3%) | 20 (23%) | 20 (23%) | 6 (6.8%) | 4 (4.5%) | 42 (48%) | 2 (2.3%) | 18 (21%) | 31 (36%) | 12 (14%) | 35 (40%) | 39 (44%) | 11 (12%) | 1 (1.1%) | 5 (5.7%) |
| 4 Excellent - Both proximal and distal portion identified | 0 (0%) | 7 (8.0%) | 61 (70%) | 0 (0%) | 0 (0%) | 16 (18%) | 1 (1.1%) | 3 (3.4%) | 36 (41%) | 2 (2.3%) | 30 (34%) | 15 (17%) | 9 (10%) | 1 (1.1%) | 1 (1.1%) |
| Not within FOV | 0 (0%) | 7 (8.0%) | 0 (0%) | 0 (0%) | 0 (0%) | 0 (0%) | 6 (6.8%) | 7 (8.0%) | 0 (0%) | 0 (0%) | 1 (1.1%) | 25 (28%) | 25 (28%) | 61 (69%) | 6 (6.8%) |
| **SI proximal** |  |  |  |  |  |  |  |  |  |  |  |  |  |  |  |
| N | 3 | 30 | 43 | 10 | 13 | 44 | 7 | 36 | 43 | 15 | 43 | 31 | 17 | 14 | 13 |
| Mean (SD) | 642 (293) | 685 (176) | 623 (130) | 621 (201) | 545 (130) | 557 (122) | 645 (351) | 974 (300) | 584 (128) | 581 (124) | 664 (168) | 598 (228) | 611 (194) | 663 (186) | 598 (180) |
| Median (IQR) | 598 (485, 776) | 670 (549, 778) | 634 (518, 693) | 625 (527, 672) | 545 (460, 585) | 562 (473, 618) | 562 (405, 903) | 935 (815, 1,200) | 558 (504, 646) | 626 (490, 667) | 651 (529, 790) | 583 (431, 747) | 557 (489, 724) | 706 (526, 829) | 633 (448, 720) |
| Range | 373, 954 | 339, 1,159 | 298, 864 | 264, 1,042 | 344, 816 | 297, 854 | 210, 1,126 | 424, 1,564 | 279, 878 | 346, 774 | 267, 961 | 267, 1,353 | 308, 980 | 356, 917 | 266, 876 |
| **SI mid** |  |  |  |  |  |  |  |  |  |  |  |  |  |  |  |
| N | 1 | 16 | 42 | 7 | 1 | 38 | 0 | 15 | 39 | 8 | 39 | 29 | 10 | 6 | 5 |
| Mean (SD) | 735 (NA) | 535 (224) | 538 (167) | 682 (252) | 599 (NA) | 545 (139) | NA (NA) | 655 (263) | 519 (153) | 435 (130) | 626 (146) | 460 (172) | 466 (178) | 546 (182) | 396 (117) |
| Median (IQR) | 735 (735, 735) | 542 (434, 593) | 533 (451, 618) | 684 (549, 738) | 599 (599, 599) | 537 (437, 636) | NA (NA, NA) | 693 (494, 786) | 517 (408, 593) | 415 (344, 529) | 632 (550, 710) | 427 (381, 529) | 451 (369, 611) | 558 (443, 665) | 348 (330, 475) |
| Range | 735, 735 | 0, 933 | 224, 1,137 | 373, 1,143 | 599, 599 | 289, 990 | Inf, -Inf | 1, 1,041 | 269, 946 | 270, 648 | 192, 1,057 | 0, 906 | 125, 699 | 278, 779 | 269, 557 |
| **SI distal** |  |  |  |  |  |  |  |  |  |  |  |  |  |  |  |
| N | 2 | 12 | 39 | 4 | 1 | 32 | 0 | 11 | 31 | 8 | 36 | 25 | 8 | 2 | 5 |
| Mean (SD) | 254 (360) | 510 (152) | 337 (132) | 581 (208) | 382 (NA) | 584 (164) | NA (NA) | 432 (191) | 446 (146) | 386 (96) | 443 (141) | 401 (114) | 377 (126) | 245 (60) | 321 (193) |
| Median (IQR) | 254 (127, 382) | 474 (429, 536) | 339 (226, 447) | 633 (471, 743) | 382 (382, 382) | 560 (467, 701) | NA (NA, NA) | 415 (383, 511) | 445 (342, 528) | 416 (303, 427) | 419 (349, 549) | 368 (316, 473) | 407 (331, 450) | 245 (223, 266) | 361 (315, 422) |
| Range | 0, 509 | 334, 813 | 148, 672 | 310, 745 | 382, 382 | 280, 966 | Inf, -Inf | 0, 782 | 195, 778 | 256, 547 | 64, 758 | 183, 684 | 112, 511 | 202, 287 | 0, 507 |
| **Diameter proximal** |  |  |  |  |  |  |  |  |  |  |  |  |  |  |  |
| N | 3 | 30 | 43 | 10 | 13 | 44 | 7 | 35 | 43 | 15 | 43 | 30 | 17 | 14 | 13 |
| Mean (SD) | 1.17 (0.36) | 1.17 (0.25) | 1.64 (0.42) | 1.32 (0.25) | 1.23 (0.23) | 1.17 (0.20) | 1.09 (0.20) | 1.27 (0.24) | 1.27 (0.30) | 1.20 (0.25) | 1.81 (0.53) | 1.32 (0.31) | 1.40 (0.47) | 1.21 (0.26) | 1.34 (0.20) |
| Median (IQR) | 1.10 (0.98, 1.33) | 1.10 (1.00, 1.32) | 1.68 (1.34, 1.94) | 1.19 (1.12, 1.56) | 1.20 (1.08, 1.42) | 1.16 (1.02, 1.36) | 1.03 (0.96, 1.14) | 1.22 (1.12, 1.37) | 1.21 (1.08, 1.39) | 1.21 (0.97, 1.36) | 1.70 (1.48, 2.13) | 1.34 (1.12, 1.55) | 1.28 (1.02, 1.64) | 1.19 (1.02, 1.33) | 1.30 (1.20, 1.51) |
| Range | 0.86, 1.56 | 0.85, 1.90 | 0.91, 2.80 | 1.06, 1.76 | 0.87, 1.57 | 0.80, 1.49 | 0.91, 1.48 | 0.90, 2.10 | 0.80, 2.40 | 0.86, 1.60 | 0.89, 3.00 | 0.68, 2.06 | 0.88, 2.57 | 0.80, 1.74 | 1.08, 1.78 |
| **Diameter mid** |  |  |  |  |  |  |  |  |  |  |  |  |  |  |  |
| N | 1 | 15 | 42 | 7 | 1 | 38 | 0 | 14 | 39 | 8 | 39 | 27 | 10 | 6 | 5 |
| Mean (SD) | 1.20 (NA) | 1.06 (0.18) | 1.96 (0.41) | 1.20 (0.43) | 1.01 (NA) | 1.12 (0.24) | NA (NA) | 1.26 (0.25) | 1.23 (0.33) | 1.20 (0.30) | 1.64 (0.40) | 1.17 (0.43) | 1.20 (0.27) | 1.14 (0.36) | 1.23 (0.18) |
| Median (IQR) | 1.20 (1.20, 1.20) | 1.01 (0.93, 1.16) | 2.00 (1.72, 2.22) | 1.20 (1.16, 1.28) | 1.01 (1.01, 1.01) | 1.16 (0.94, 1.27) | NA (NA, NA) | 1.29 (1.10, 1.43) | 1.15 (0.99, 1.56) | 1.15 (0.96, 1.44) | 1.67 (1.34, 1.89) | 1.09 (0.92, 1.26) | 1.12 (1.00, 1.31) | 1.17 (0.89, 1.21) | 1.33 (1.22, 1.33) |
| Range | 1.20, 1.20 | 0.81, 1.52 | 1.18, 2.90 | 0.43, 1.90 | 1.01, 1.01 | 0.70, 1.80 | Inf, -Inf | 0.82, 1.70 | 0.60, 1.75 | 0.82, 1.65 | 0.82, 2.37 | 0.81, 3.03 | 0.90, 1.70 | 0.75, 1.76 | 0.91, 1.33 |
| **Diameter distal** |  |  |  |  |  |  |  |  |  |  |  |  |  |  |  |
| N | 1 | 12 | 39 | 4 | 1 | 32 | 0 | 10 | 31 | 8 | 36 | 25 | 8 | 2 | 4 |
| Mean (SD) | 1.10 (NA) | 0.95 (0.20) | 1.73 (0.43) | 1.16 (0.49) | 1.03 (NA) | 0.97 (0.23) | NA (NA) | 1.20 (0.29) | 1.02 (0.23) | 0.94 (0.19) | 1.32 (0.41) | 1.13 (0.22) | 1.11 (0.28) | 0.65 (0.08) | 1.29 (0.58) |
| Median (IQR) | 1.10 (1.10, 1.10) | 0.97 (0.85, 1.10) | 1.59 (1.42, 2.04) | 1.21 (0.96, 1.42) | 1.03 (1.03, 1.03) | 0.91 (0.77, 1.16) | NA (NA, NA) | 1.07 (1.00, 1.34) | 1.04 (0.85, 1.19) | 0.92 (0.81, 1.02) | 1.34 (1.02, 1.55) | 1.15 (0.97, 1.30) | 1.08 (0.88, 1.32) | 0.65 (0.63, 0.68) | 1.18 (0.84, 1.64) |
| Range | 1.10, 1.10 | 0.58, 1.22 | 1.11, 3.02 | 0.52, 1.70 | 1.03, 1.03 | 0.64, 1.47 | Inf, -Inf | 0.92, 1.79 | 0.45, 1.48 | 0.70, 1.29 | 0.59, 2.51 | 0.75, 1.54 | 0.80, 1.54 | 0.60, 0.71 | 0.80, 2.01 |
| **aSNR proximal** |  |  |  |  |  |  |  |  |  |  |  |  |  |  |  |
| N | 3 | 30 | 43 | 10 | 13 | 44 | 7 | 36 | 43 | 15 | 43 | 31 | 17 | 14 | 13 |
| Mean (SD) | 48 (22) | 51 (13) | 47 (10) | 46 (15) | 41 (10) | 42 (9) | 48 (26) | 73 (22) | 44 (10) | 43 (9) | 50 (13) | 45 (17) | 46 (15) | 50 (14) | 45 (13) |
| Median (IQR) | 45 (36, 58) | 50 (41, 58) | 47 (39, 52) | 47 (39, 50) | 41 (34, 44) | 42 (35, 46) | 42 (30, 68) | 70 (61, 90) | 42 (38, 48) | 47 (37, 50) | 49 (40, 59) | 44 (32, 56) | 42 (37, 54) | 53 (39, 62) | 47 (33, 54) |
| Range | 28, 71 | 25, 87 | 22, 65 | 20, 78 | 26, 61 | 22, 64 | 16, 84 | 32, 117 | 21, 66 | 26, 58 | 20, 72 | 20, 101 | 23, 73 | 27, 69 | 20, 65 |
| **aSNR mid** |  |  |  |  |  |  |  |  |  |  |  |  |  |  |  |
| N | 1 | 16 | 42 | 7 | 1 | 38 | 0 | 15 | 39 | 8 | 39 | 29 | 10 | 6 | 5 |
| Mean (SD) | 55 (NA) | 40 (17) | 40 (12) | 51 (19) | 45 (NA) | 41 (10) | NA (NA) | 49 (20) | 39 (11) | 33 (10) | 47 (11) | 34 (13) | 35 (13) | 41 (14) | 30 (9) |
| Median (IQR) | 55 (55, 55) | 41 (32, 44) | 40 (34, 46) | 51 (41, 55) | 45 (45, 45) | 40 (33, 48) | NA (NA, NA) | 52 (37, 59) | 39 (30, 44) | 31 (26, 40) | 47 (41, 53) | 32 (29, 40) | 34 (28, 46) | 42 (33, 50) | 26 (25, 35) |
| Range | 55, 55 | 0, 70 | 17, 85 | 28, 85 | 45, 45 | 22, 74 | Inf, -Inf | 0, 78 | 20, 71 | 20, 48 | 14, 79 | 0, 68 | 9, 52 | 21, 58 | 20, 42 |
| **aSNR distal** |  |  |  |  |  |  |  |  |  |  |  |  |  |  |  |
| N | 2 | 12 | 39 | 4 | 1 | 32 | 0 | 11 | 31 | 8 | 36 | 25 | 8 | 2 | 5 |
| Mean (SD) | 19 (27) | 38 (11) | 25 (10) | 43 (16) | 29 (NA) | 44 (12) | NA (NA) | 32 (14) | 33 (11) | 29 (7) | 33 (11) | 30 (9) | 28 (9) | 18 (4) | 24 (14) |
| Median (IQR) | 19 (10, 29) | 35 (32, 40) | 25 (17, 33) | 47 (35, 56) | 29 (29, 29) | 42 (35, 52) | NA (NA, NA) | 31 (29, 38) | 33 (26, 39) | 31 (23, 32) | 31 (26, 41) | 27 (24, 35) | 30 (25, 34) | 18 (17, 20) | 27 (24, 32) |
| Range | 0, 38 | 25, 61 | 11, 50 | 23, 56 | 29, 29 | 21, 72 | Inf, -Inf | 0, 58 | 15, 58 | 19, 41 | 5, 57 | 14, 51 | 8, 38 | 15, 21 | 0, 38 |
| **aNMCNR proximal** |  |  |  |  |  |  |  |  |  |  |  |  |  |  |  |
| N | 3 | 30 | 43 | 10 | 13 | 44 | 7 | 36 | 43 | 15 | 43 | 31 | 17 | 14 | 13 |
| Mean (SD) | 34 (20) | 34 (13) | 30 (9) | 31 (14) | 25 (9) | 25 (9) | 32 (25) | 56 (21) | 27 (9) | 27 (8) | 33 (12) | 28 (17) | 29 (15) | 33 (17) | 28 (13) |
| Median (IQR) | 29 (23, 42) | 34 (25, 40) | 30 (22, 37) | 32 (27, 35) | 26 (18, 29) | 25 (20, 30) | 26 (15, 50) | 54 (44, 73) | 26 (20, 33) | 30 (22, 33) | 32 (24, 40) | 29 (16, 38) | 26 (21, 35) | 38 (23, 48) | 32 (18, 35) |
| Range | 17, 56 | 10, 69 | 11, 49 | 9, 62 | 14, 45 | 6, 42 | 0, 67 | 16, 93 | 10, 48 | 11, 41 | 9, 57 | 3, 86 | 6, 59 | 1, 54 | 4, 47 |
| **aNMCNR mid** |  |  |  |  |  |  |  |  |  |  |  |  |  |  |  |
| N | 1 | 16 | 42 | 7 | 1 | 38 | 0 | 15 | 39 | 8 | 39 | 29 | 10 | 6 | 5 |
| Mean (SD) | 39 (NA) | 23 (16) | 23 (11) | 35 (19) | 29 (NA) | 24 (10) | NA (NA) | 33 (19) | 22 (11) | 16 (9) | 30 (11) | 18 (12) | 19 (12) | 27 (14) | 13 (7) |
| Median (IQR) | 39 (39, 39) | 23 (16, 31) | 23 (17, 30) | 36 (26, 40) | 29 (29, 29) | 24 (16, 29) | NA (NA, NA) | 35 (25, 44) | 22 (13, 28) | 15 (11, 22) | 29 (25, 36) | 16 (13, 23) | 22 (12, 27) | 26 (17, 35) | 9 (9, 18) |
| Range | 39, 39 | -16, 49 | 5, 68 | 12, 68 | 29, 29 | 6, 54 | Inf, -Inf | -17, 61 | 7, 54 | 4, 31 | 3, 62 | -16, 48 | -6, 34 | 10, 47 | 5, 22 |
| **aNMCNR distal** |  |  |  |  |  |  |  |  |  |  |  |  |  |  |  |
| N | 2 | 12 | 39 | 4 | 1 | 32 | 0 | 11 | 31 | 8 | 36 | 25 | 8 | 2 | 5 |
| Mean (SD) | 2 (29) | 21 (10) | 8 (9) | 28 (16) | 13 (NA) | 26 (11) | NA (NA) | 16 (14) | 16 (10) | 13 (7) | 17 (11) | 14 (8) | 13 (10) | 2 (4) | 8 (14) |
| Median (IQR) | 2 (-9, 12) | 19 (15, 24) | 6 (1, 15) | 31 (19, 40) | 13 (13, 13) | 26 (19, 34) | NA (NA, NA) | 16 (13, 21) | 15 (7, 23) | 14 (8, 15) | 16 (11, 23) | 12 (9, 19) | 14 (9, 17) | 2 (0, 4) | 11 (8, 14) |
| Range | -19, 22 | 8, 43 | -5, 33 | 7, 41 | 13, 13 | 5, 54 | Inf, -Inf | -16, 41 | 3, 40 | 3, 24 | -15, 45 | 0, 33 | -6, 29 | -1, 5 | -16, 21 |
| **After gadolinium contrast administration** | | | | | | | | | | | | | | | |
| **Nerve visualisation score** |  |  |  |  |  |  |  |  |  |  |  |  |  |  |  |
| 0 Nerve not identified | 24 (27%) | 2 (2.3%) | 0 (0%) | 2 (2.3%) | 0 (0%) | 0 (0%) | 7 (8.0%) | 0 (0%) | 0 (0%) | 3 (3.4%) | 0 (0%) | 0 (0%) | 2 (2.3%) | 0 (0%) | 11 (12%) |
| 1 Poor - Only proximal portion identified but not continuous | 26 (30%) | 10 (11%) | 0 (0%) | 2 (2.3%) | 2 (2.3%) | 0 (0%) | 20 (23%) | 1 (1.1%) | 0 (0%) | 2 (2.3%) | 0 (0%) | 0 (0%) | 3 (3.4%) | 5 (5.6%) | 17 (19%) |
| 2 Fair - Only proximal portion identified | 10 (11%) | 8 (9.1%) | 2 (2.3%) | 7 (8.0%) | 9 (10%) | 0 (0%) | 22 (25%) | 13 (15%) | 2 (2.3%) | 7 (8.0%) | 7 (8.0%) | 0 (0%) | 11 (12%) | 12 (13%) | 34 (39%) |
| 3 Good Fair - Both portions identified but not continuous | 18 (20%) | 28 (32%) | 4 (4.5%) | 28 (32%) | 36 (41%) | 11 (12%) | 32 (36%) | 24 (27%) | 0 (0%) | 31 (35%) | 25 (28%) | 6 (6.8%) | 16 (18%) | 8 (9.0%) | 23 (26%) |
| 4 Excellent - Both proximal and distal portion identified | 10 (11%) | 37 (42%) | 82 (93%) | 49 (56%) | 41 (47%) | 77 (88%) | 5 (5.7%) | 46 (52%) | 85 (98%) | 45 (51%) | 56 (64%) | 58 (66%) | 20 (23%) | 7 (7.9%) | 1 (1.1%) |
| Not within FOV | 0 (0%) | 3 (3.4%) | 0 (0%) | 0 (0%) | 0 (0%) | 0 (0%) | 2 (2.3%) | 4 (4.5%) | 0 (0%) | 0 (0%) | 0 (0%) | 24 (27%) | 36 (41%) | 57 (64%) | 2 (2.3%) |
| **SI proximal** |  |  |  |  |  |  |  |  |  |  |  |  |  |  |  |
| N | 31 | 41 | 44 | 43 | 44 | 44 | 38 | 42 | 43 | 42 | 44 | 32 | 22 | 15 | 35 |
| Mean (SD) | 392 (143) | 563 (164) | 619 (125) | 506 (106) | 493 (117) | 506 (183) | 529 (231) | 921 (322) | 562 (146) | 530 (147) | 632 (170) | 592 (242) | 593 (176) | 769 (187) | 614 (207) |
| Median (IQR) | 337 (277, 497) | 580 (456, 677) | 588 (519, 701) | 499 (440, 583) | 479 (421, 572) | 458 (371, 559) | 489 (359, 614) | 916 (658, 1,132) | 553 (446, 664) | 530 (422, 603) | 592 (501, 738) | 616 (408, 761) | 638 (484, 727) | 741 (614, 894) | 582 (485, 728) |
| Range | 222, 727 | 188, 870 | 420, 865 | 264, 709 | 306, 727 | 248, 1,075 | 256, 1,202 | 249, 1,635 | 346, 907 | 319, 963 | 350, 1,051 | 173, 1,000 | 196, 812 | 569, 1,244 | 200, 1,168 |
| **SI mid** |  |  |  |  |  |  |  |  |  |  |  |  |  |  |  |
| N | 14 | 32 | 44 | 40 | 40 | 44 | 21 | 39 | 43 | 42 | 42 | 32 | 18 | 12 | 16 |
| Mean (SD) | 278 (61) | 462 (139) | 507 (124) | 467 (124) | 409 (101) | 495 (130) | 423 (148) | 590 (173) | 469 (105) | 428 (117) | 571 (147) | 392 (136) | 518 (242) | 647 (120) | 479 (152) |
| Median (IQR) | 265 (237, 295) | 444 (395, 537) | 484 (424, 571) | 479 (364, 545) | 397 (348, 460) | 489 (401, 566) | 433 (363, 528) | 576 (474, 732) | 450 (392, 556) | 375 (344, 512) | 546 (485, 682) | 364 (306, 496) | 512 (332, 718) | 635 (571, 723) | 475 (371, 548) |
| Range | 189, 387 | 156, 796 | 289, 832 | 261, 712 | 244, 652 | 265, 836 | 1, 637 | 221, 997 | 289, 747 | 266, 794 | 253, 920 | 136, 731 | 103, 886 | 479, 897 | 261, 837 |
| **SI distal** |  |  |  |  |  |  |  |  |  |  |  |  |  |  |  |
| N | 10 | 32 | 41 | 40 | 38 | 44 | 14 | 35 | 41 | 39 | 40 | 31 | 16 | 9 | 8 |
| Mean (SD) | 299 (130) | 370 (115) | 301 (111) | 411 (136) | 320 (58) | 444 (150) | 357 (66) | 371 (155) | 337 (110) | 346 (84) | 387 (136) | 386 (134) | 367 (168) | 332 (132) | 375 (96) |
| Median (IQR) | 259 (241, 304) | 361 (303, 416) | 307 (214, 364) | 431 (343, 492) | 320 (281, 351) | 440 (315, 549) | 361 (322, 384) | 352 (272, 464) | 334 (263, 386) | 338 (298, 397) | 370 (299, 472) | 353 (331, 463) | 348 (248, 457) | 307 (296, 326) | 346 (315, 382) |
| Range | 152, 626 | 131, 681 | 139, 729 | 163, 722 | 199, 435 | 177, 813 | 241, 477 | 0, 818 | 174, 674 | 180, 553 | 188, 772 | 146, 692 | 103, 741 | 189, 663 | 290, 572 |
| **Diameter proximal** |  |  |  |  |  |  |  |  |  |  |  |  |  |  |  |
| N | 31 | 41 | 44 | 43 | 44 | 44 | 37 | 42 | 43 | 42 | 44 | 32 | 22 | 15 | 35 |
| Mean (SD) | 0.93 (0.27) | 1.13 (0.26) | 1.78 (0.44) | 1.13 (0.24) | 1.14 (0.24) | 1.24 (0.25) | 1.08 (0.23) | 1.28 (0.29) | 1.38 (0.34) | 1.18 (0.25) | 1.78 (0.56) | 1.42 (0.37) | 1.42 (0.26) | 1.37 (0.38) | 1.25 (0.36) |
| Median (IQR) | 0.89 (0.72, 1.09) | 1.10 (0.98, 1.23) | 1.68 (1.48, 2.06) | 1.16 (0.95, 1.27) | 1.13 (0.96, 1.28) | 1.22 (1.05, 1.43) | 1.09 (0.90, 1.24) | 1.23 (1.12, 1.35) | 1.31 (1.13, 1.52) | 1.12 (1.03, 1.29) | 1.67 (1.37, 2.12) | 1.32 (1.18, 1.69) | 1.35 (1.27, 1.53) | 1.25 (1.10, 1.54) | 1.16 (1.03, 1.49) |
| Range | 0.50, 1.48 | 0.60, 2.00 | 1.00, 2.86 | 0.64, 1.69 | 0.65, 1.74 | 0.60, 1.79 | 0.60, 1.59 | 0.80, 2.46 | 0.80, 2.36 | 0.76, 2.20 | 0.90, 2.95 | 0.84, 2.45 | 1.10, 2.11 | 0.91, 2.13 | 0.60, 2.18 |
| **Diameter mid** |  |  |  |  |  |  |  |  |  |  |  |  |  |  |  |
| N | 14 | 32 | 44 | 40 | 40 | 44 | 20 | 39 | 43 | 42 | 42 | 32 | 18 | 12 | 16 |
| Mean (SD) | 0.75 (0.17) | 0.98 (0.23) | 1.68 (0.49) | 1.10 (0.25) | 1.02 (0.22) | 1.18 (0.21) | 1.04 (0.17) | 1.12 (0.17) | 1.22 (0.30) | 1.09 (0.18) | 1.62 (0.40) | 1.22 (0.23) | 1.24 (0.29) | 1.25 (0.31) | 1.10 (0.28) |
| Median (IQR) | 0.74 (0.61, 0.80) | 0.94 (0.82, 1.17) | 1.57 (1.35, 1.77) | 1.05 (0.90, 1.24) | 1.00 (0.90, 1.16) | 1.17 (1.03, 1.30) | 1.01 (0.90, 1.14) | 1.10 (1.00, 1.23) | 1.14 (1.06, 1.38) | 1.06 (1.00, 1.18) | 1.56 (1.33, 1.89) | 1.19 (1.08, 1.38) | 1.22 (1.01, 1.37) | 1.23 (1.00, 1.37) | 1.02 (0.93, 1.19) |
| Range | 0.50, 1.13 | 0.55, 1.58 | 1.14, 3.33 | 0.66, 1.66 | 0.54, 1.55 | 0.75, 1.69 | 0.76, 1.34 | 0.76, 1.50 | 0.60, 2.27 | 0.78, 1.59 | 1.00, 2.80 | 0.72, 1.79 | 0.89, 2.00 | 0.84, 1.97 | 0.70, 1.85 |
| **Diameter distal** |  |  |  |  |  |  |  |  |  |  |  |  |  |  |  |
| N | 10 | 32 | 41 | 40 | 38 | 44 | 14 | 34 | 41 | 39 | 40 | 32 | 16 | 9 | 7 |
| Mean (SD) | 0.69 (0.13) | 0.97 (0.19) | 1.31 (0.30) | 0.97 (0.21) | 0.89 (0.22) | 1.05 (0.20) | 0.95 (0.23) | 1.15 (0.20) | 1.08 (0.21) | 0.90 (0.19) | 1.15 (0.28) | 1.16 (0.23) | 1.00 (0.18) | 1.00 (0.18) | 1.02 (0.31) |
| Median (IQR) | 0.66 (0.60, 0.77) | 0.96 (0.84, 1.10) | 1.27 (1.06, 1.54) | 0.96 (0.85, 1.09) | 0.89 (0.73, 1.02) | 1.06 (0.91, 1.20) | 0.96 (0.82, 1.01) | 1.14 (1.01, 1.30) | 1.05 (0.94, 1.25) | 0.87 (0.80, 0.97) | 1.12 (0.96, 1.36) | 1.21 (1.06, 1.31) | 1.00 (0.83, 1.12) | 0.93 (0.90, 1.12) | 0.99 (0.83, 1.07) |
| Range | 0.50, 0.90 | 0.61, 1.37 | 0.82, 2.15 | 0.61, 1.46 | 0.45, 1.28 | 0.62, 1.49 | 0.60, 1.41 | 0.70, 1.51 | 0.70, 1.55 | 0.60, 1.42 | 0.67, 1.89 | 0.51, 1.53 | 0.74, 1.31 | 0.68, 1.30 | 0.71, 1.63 |
| **aSNR proximal** |  |  |  |  |  |  |  |  |  |  |  |  |  |  |  |
| N | 31 | 41 | 44 | 43 | 44 | 44 | 38 | 42 | 43 | 42 | 44 | 32 | 22 | 15 | 35 |
| Mean (SD) | 29 (11) | 42 (12) | 46 (9) | 38 (8) | 37 (9) | 38 (14) | 40 (17) | 69 (24) | 42 (11) | 40 (11) | 47 (13) | 44 (18) | 44 (13) | 57 (14) | 46 (15) |
| Median (IQR) | 25 (21, 37) | 43 (34, 51) | 44 (39, 52) | 37 (33, 44) | 36 (31, 43) | 34 (28, 42) | 37 (27, 46) | 68 (49, 85) | 41 (33, 50) | 40 (32, 45) | 44 (37, 55) | 46 (30, 57) | 48 (36, 54) | 55 (46, 67) | 44 (36, 54) |
| Range | 17, 54 | 14, 65 | 31, 65 | 20, 53 | 23, 54 | 19, 80 | 19, 90 | 19, 122 | 26, 68 | 24, 72 | 26, 79 | 13, 75 | 15, 61 | 43, 93 | 15, 87 |
| **aSNR mid** |  |  |  |  |  |  |  |  |  |  |  |  |  |  |  |
| N | 14 | 32 | 44 | 40 | 40 | 44 | 21 | 39 | 43 | 42 | 42 | 32 | 18 | 12 | 16 |
| Mean (SD) | 21 (5) | 35 (10) | 38 (9) | 35 (9) | 31 (8) | 37 (10) | 32 (11) | 44 (13) | 35 (8) | 32 (9) | 43 (11) | 29 (10) | 39 (18) | 48 (9) | 36 (11) |
| Median (IQR) | 20 (18, 22) | 33 (30, 40) | 36 (32, 43) | 36 (27, 41) | 30 (26, 34) | 37 (30, 42) | 32 (27, 39) | 43 (35, 55) | 34 (29, 42) | 28 (26, 38) | 41 (36, 51) | 27 (23, 37) | 38 (25, 54) | 47 (43, 54) | 36 (28, 41) |
| Range | 14, 29 | 12, 60 | 22, 62 | 19, 53 | 18, 49 | 20, 63 | 0, 48 | 16, 75 | 22, 56 | 20, 59 | 19, 69 | 10, 55 | 8, 66 | 36, 67 | 20, 63 |
| **aSNR distal** |  |  |  |  |  |  |  |  |  |  |  |  |  |  |  |
| N | 10 | 32 | 41 | 40 | 38 | 44 | 14 | 35 | 41 | 39 | 40 | 31 | 16 | 9 | 8 |
| Mean (SD) | 22 (10) | 28 (9) | 23 (8) | 31 (10) | 24 (4) | 33 (11) | 27 (5) | 28 (12) | 25 (8) | 26 (6) | 29 (10) | 29 (10) | 27 (13) | 25 (10) | 28 (7) |
| Median (IQR) | 19 (18, 23) | 27 (23, 31) | 23 (16, 27) | 32 (26, 37) | 24 (21, 26) | 33 (24, 41) | 27 (24, 29) | 26 (20, 35) | 25 (20, 29) | 25 (22, 30) | 28 (22, 35) | 26 (25, 35) | 26 (19, 34) | 23 (22, 24) | 26 (24, 29) |
| Range | 11, 47 | 10, 51 | 10, 54 | 12, 54 | 15, 32 | 13, 61 | 18, 36 | 0, 61 | 13, 50 | 13, 41 | 14, 58 | 11, 52 | 8, 55 | 14, 50 | 22, 43 |
| **aNMCNR proximal** |  |  |  |  |  |  |  |  |  |  |  |  |  |  |  |
| N | 31 | 41 | 44 | 43 | 44 | 44 | 38 | 42 | 43 | 42 | 44 | 32 | 22 | 15 | 35 |
| Mean (SD) | 17 (11) | 30 (12) | 34 (9) | 26 (8) | 25 (9) | 26 (14) | 27 (17) | 57 (24) | 30 (11) | 28 (11) | 35 (13) | 32 (18) | 31 (13) | 46 (15) | 34 (16) |
| Median (IQR) | 13 (9, 25) | 32 (21, 39) | 32 (28, 40) | 25 (20, 31) | 23 (18, 30) | 23 (17, 29) | 24 (13, 36) | 58 (37, 71) | 29 (21, 38) | 25 (20, 33) | 33 (25, 44) | 36 (16, 45) | 32 (24, 41) | 46 (34, 56) | 30 (24, 42) |
| Range | 5, 45 | 0, 54 | 20, 55 | 9, 41 | 11, 43 | -1, 71 | 6, 76 | 4, 110 | 8, 55 | 12, 60 | 17, 69 | 1, 62 | 0, 47 | 30, 82 | 3, 78 |
| **aNMCNR mid** |  |  |  |  |  |  |  |  |  |  |  |  |  |  |  |
| N | 14 | 32 | 44 | 40 | 40 | 44 | 21 | 39 | 43 | 42 | 42 | 32 | 18 | 12 | 16 |
| Mean (SD) | 9 (4) | 23 (11) | 26 (9) | 23 (9) | 19 (7) | 25 (9) | 19 (11) | 32 (13) | 23 (8) | 20 (9) | 31 (11) | 17 (10) | 25 (19) | 37 (9) | 24 (12) |
| Median (IQR) | 8 (6, 13) | 23 (17, 28) | 25 (20, 29) | 22 (16, 29) | 19 (14, 22) | 24 (18, 30) | 21 (13, 27) | 31 (24, 42) | 22 (18, 27) | 16 (15, 26) | 28 (23, 39) | 16 (10, 25) | 26 (11, 41) | 37 (31, 41) | 22 (16, 29) |
| Range | 3, 18 | -3, 47 | 10, 50 | 8, 44 | 5, 35 | 8, 51 | -11, 38 | 2, 62 | 2, 43 | 7, 46 | 7, 58 | -4, 42 | -7, 54 | 24, 58 | 10, 53 |
| **aNMCNR distal** |  |  |  |  |  |  |  |  |  |  |  |  |  |  |  |
| N | 10 | 32 | 41 | 40 | 38 | 44 | 14 | 35 | 41 | 39 | 40 | 31 | 16 | 9 | 8 |
| Mean (SD) | 11 (9) | 16 (8) | 10 (9) | 19 (10) | 12 (4) | 21 (11) | 14 (6) | 15 (12) | 13 (8) | 14 (7) | 17 (11) | 16 (10) | 14 (14) | 13 (11) | 16 (7) |
| Median (IQR) | 9 (7, 11) | 16 (11, 19) | 10 (3, 15) | 20 (11, 25) | 12 (9, 15) | 20 (14, 29) | 14 (10, 18) | 16 (6, 22) | 12 (7, 17) | 14 (11, 18) | 15 (9, 23) | 15 (10, 23) | 13 (4, 21) | 12 (8, 12) | 12 (12, 17) |
| Range | 0, 34 | -4, 36 | -4, 42 | 1, 41 | 3, 18 | 1, 50 | 4, 25 | -11, 48 | 1, 38 | -6, 32 | 3, 49 | -3, 39 | -7, 43 | 2, 41 | 9, 29 |
| SI: signal intensity; aSNR: apparant signal-to-noise ratio; aNMCNR: apparent nerve-muscle contrast-to-noise ratio; SD: standard deviation; IQR: interquartile range | | | | | | | | | | | | | | | |

Supplemental table 3. Benchmarking values describing nerve visualization scores, diameters, signal intensities (SI), apparent signal to noise ratio (aSNR), apparent nerve-muscle contrast-to-noise ratio (aNMCNR) measured at a proximal, mid and distal landmark for each evaluated cranial and occipital nerves. Values are given before and after gadolinium contrast administration.

Supplemental figure 1a. Apparent signal-to-noise ratio (aSNR) boxplots comparing overall proximal, middle and distal aSNR. ns: not significant, * p < 0.001.


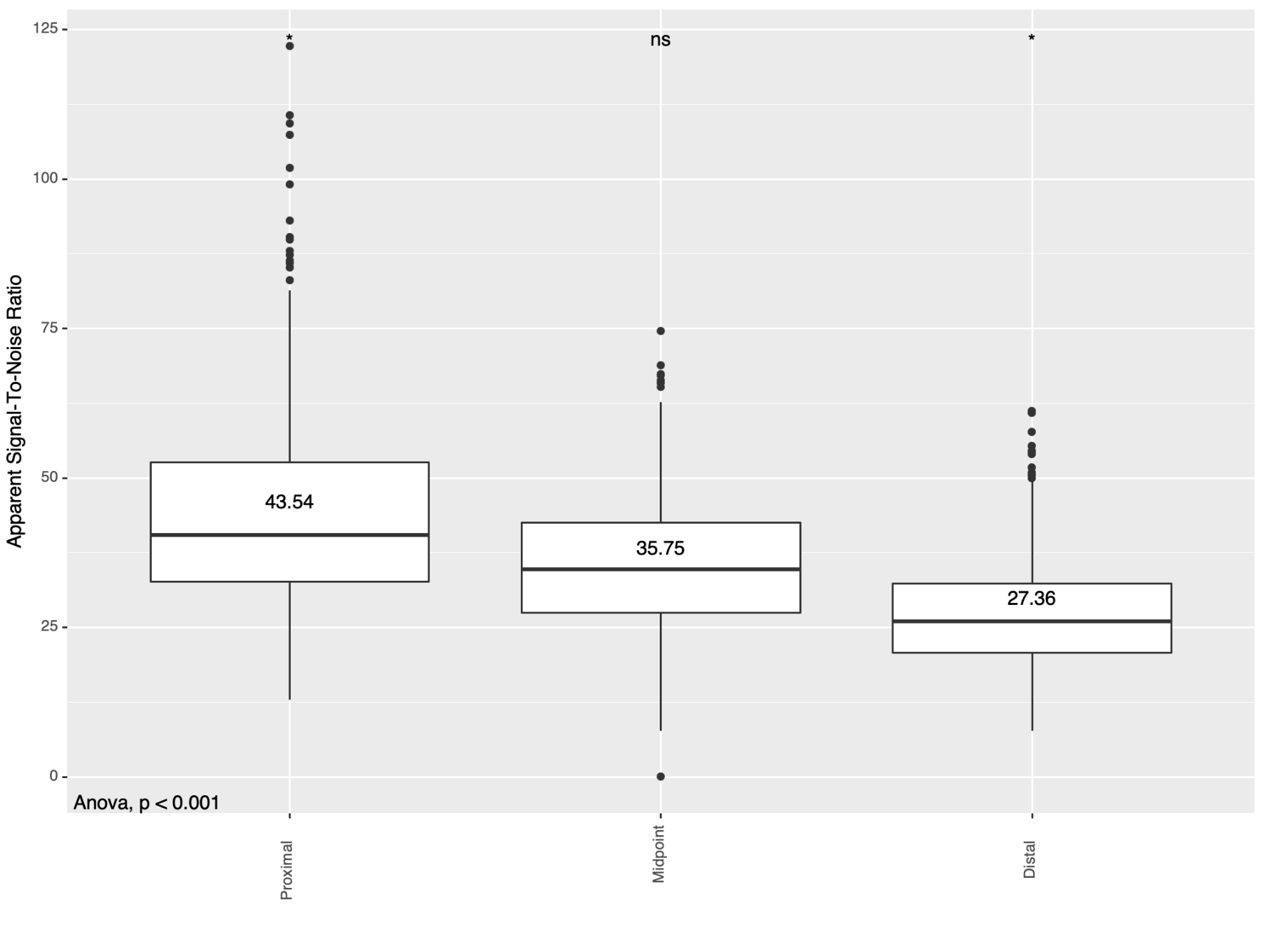


Supplemental figure 1b. Apparent signal-to-noise ratio (aSNR) boxplots comparing proximal, middle and distal aSNR, stratified according to the observed nerves.


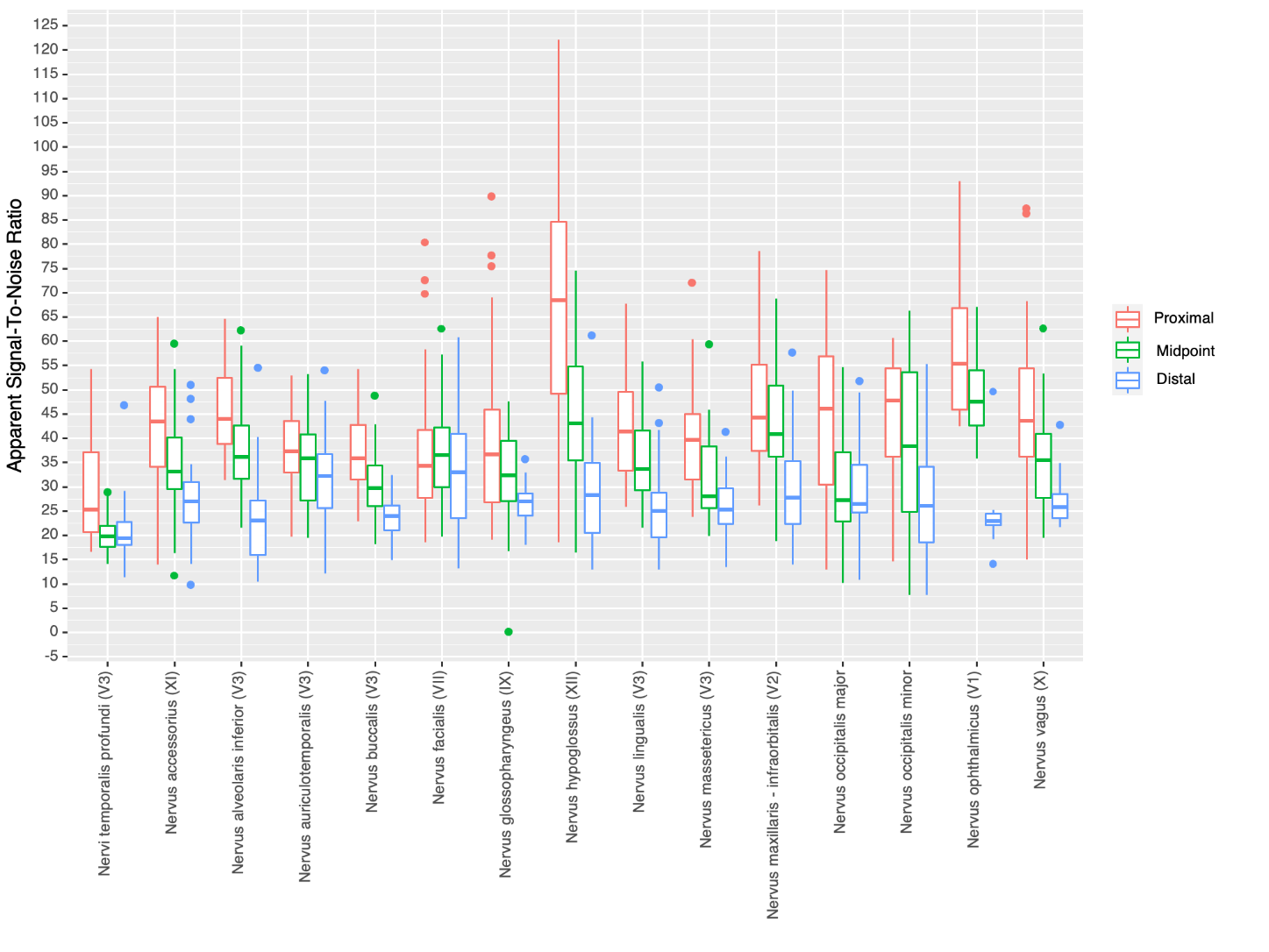


Supplemental figure 2a. Apparent nerve-muscle contrast-to-noise ratio (aNMCNR) boxplots comparing overall proximal, middle and distal aNMCNR. ns: not significant, * p < 0.001.


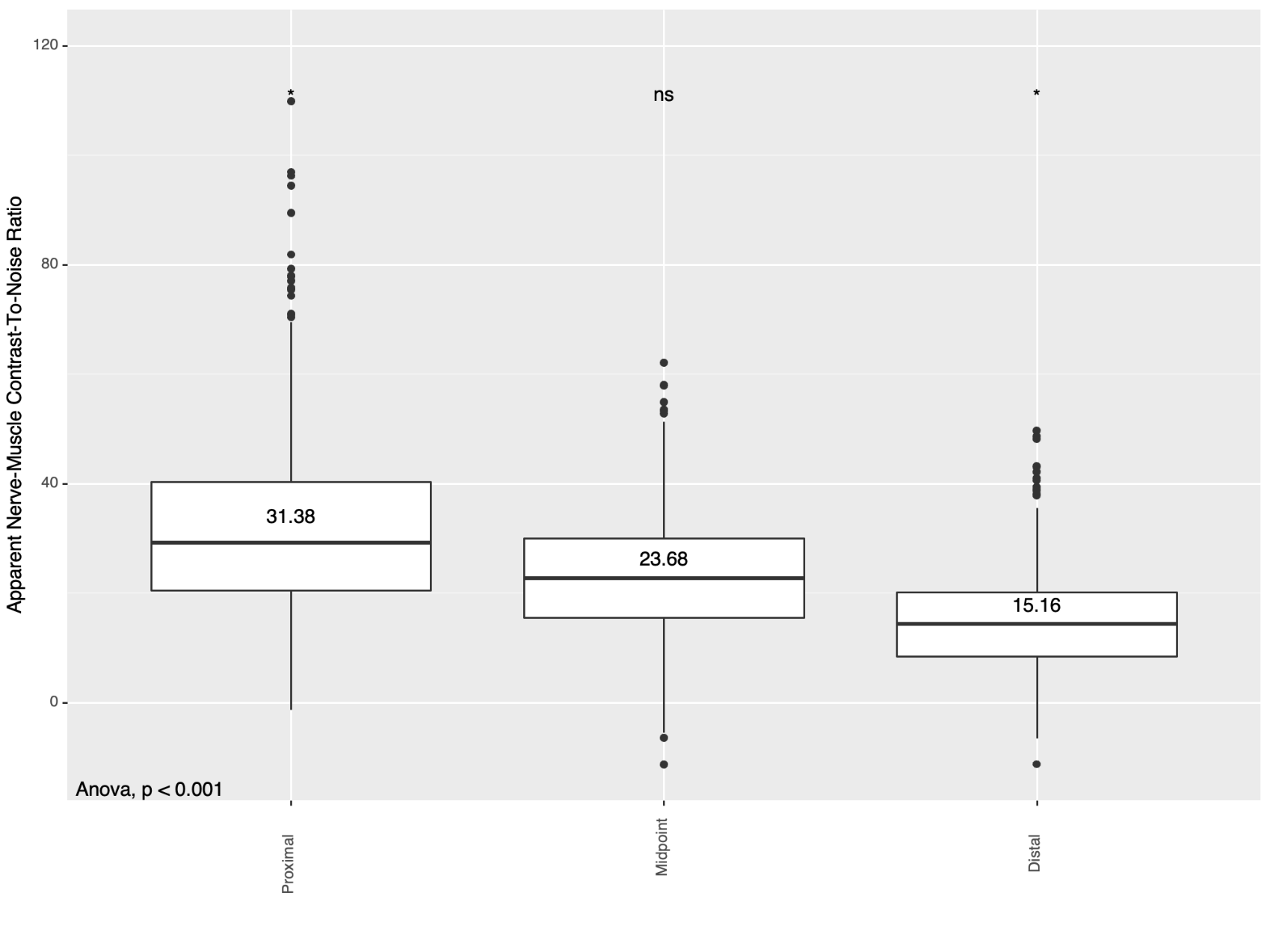


Supplemental figure 2b. Apparent nerve-muscle contrast-to-noise ratio (aNMCNR) boxplots comparing proximal, middle and distal aNMCNR, stratified according to the observed nerves.


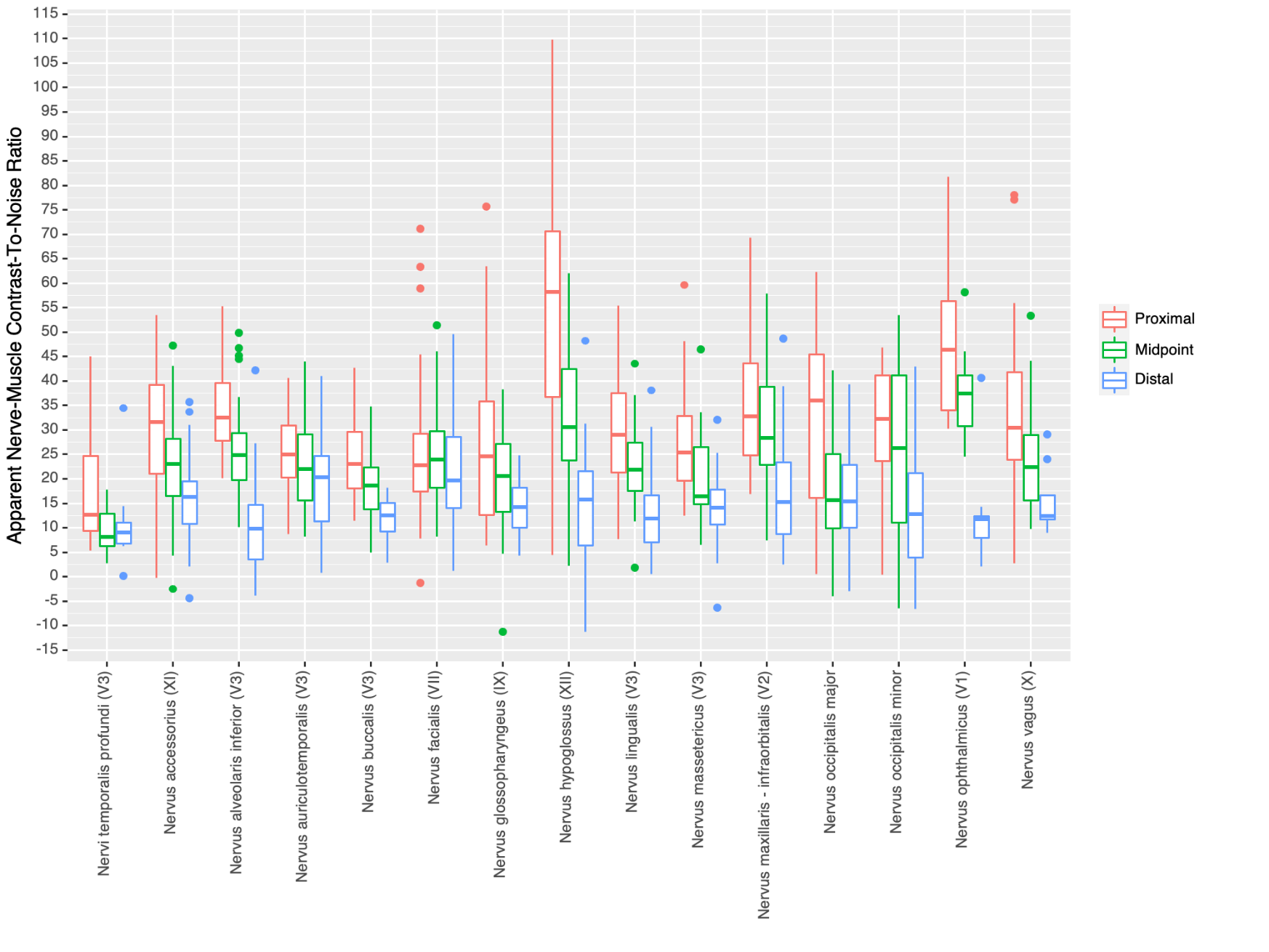


Supplemental figure 3a. Nerve diameter boxplots comparing overall proximal, middle and distal nerve diameters. ns: not significant, * p < 0.001.


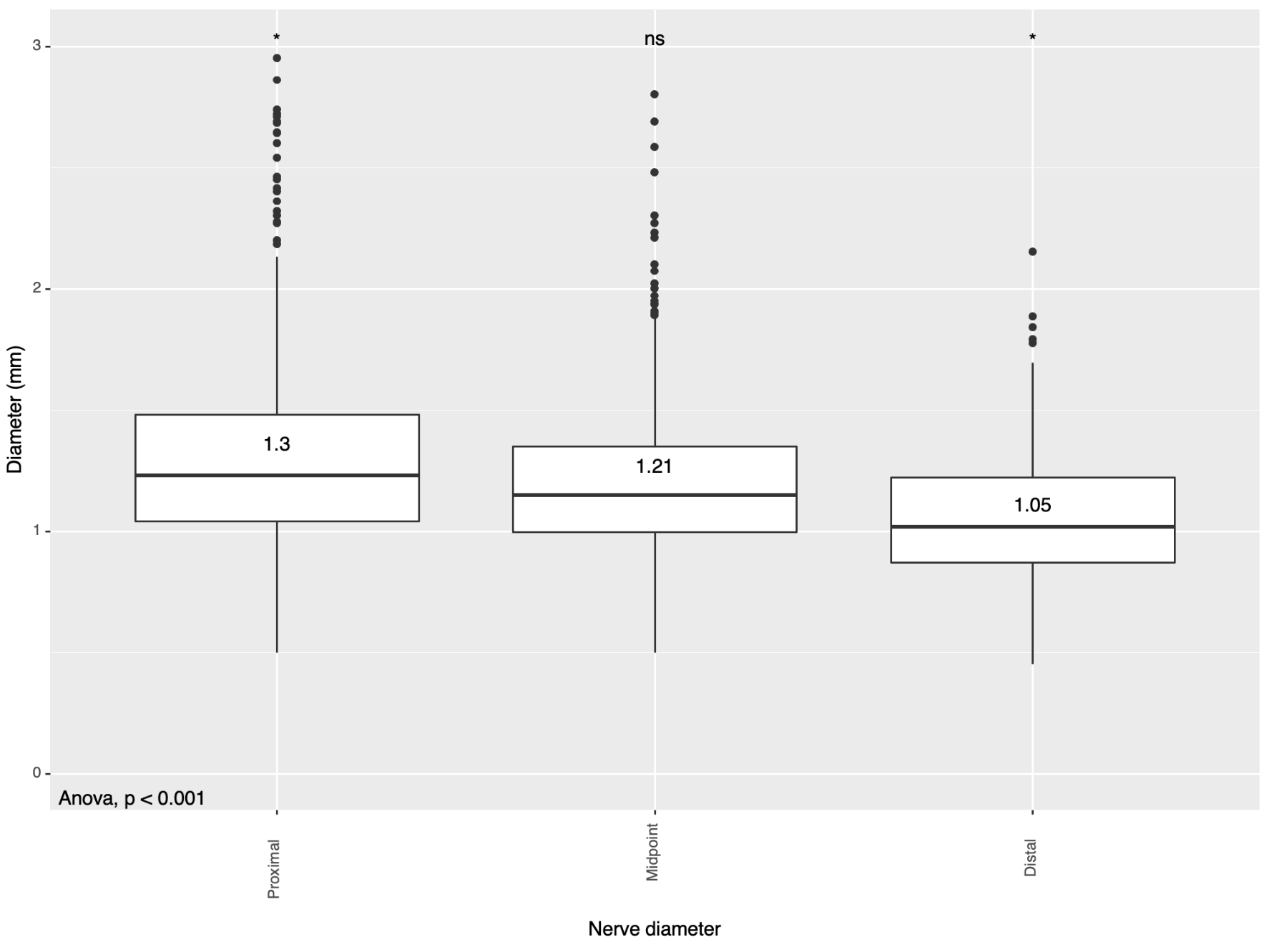


Supplemental figure 3b. Nerve diameter boxplots comparing proximal, middle and distal nerve diameters, stratified according to the observed nerves.


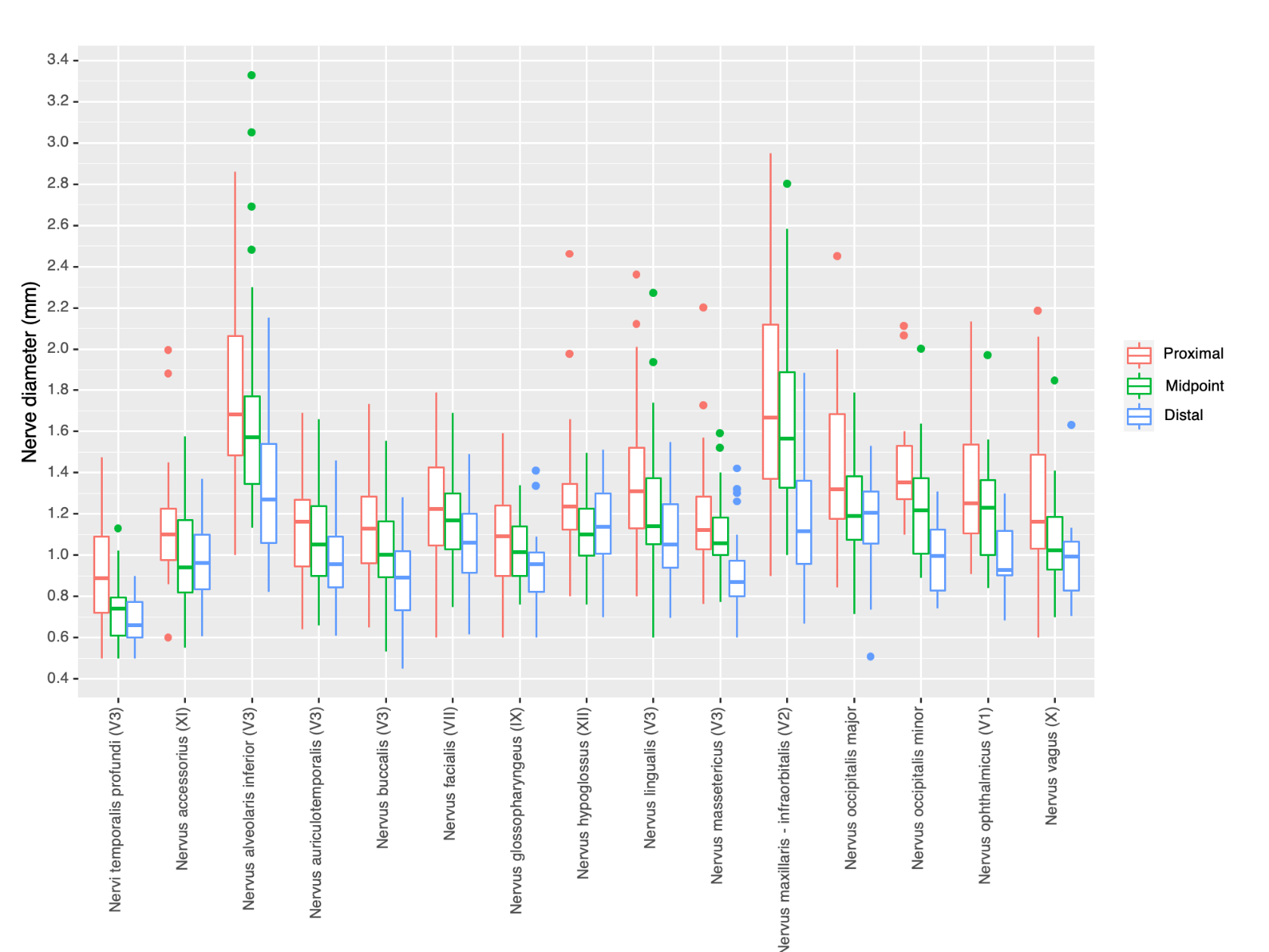

Supplement: Supplementary file 1 — (DOCX 1963 kb) [file 330_2022_9269_MOESM1_ESM.docx]
